# Targeting myeloid cell coagulation signaling blocks MAP kinase/TGF-β1–driven fibrotic remodeling in ischemic heart failure

**DOI:** 10.1172/JCI156436

**Published:** 2023-02-15

**Authors:** Venkata Garlapati, Michael Molitor, Thomas Michna, Gregory S. Harms, Stefanie Finger, Rebecca Jung, Jeremy Lagrange, Panagiotis Efentakis, Johannes Wild, Maike Knorr, Susanne Karbach, Sabine Wild, Ksenija Vujacic-Mirski, Thomas Münzel, Andreas Daiber, Moritz Brandt, Tommaso Gori, Hendrik Milting, Stefan Tenzer, Wolfram Ruf, Philip Wenzel

**Affiliations:** 1 Center for Thrombosis and Hemostasis and; 2 Department of Cardiology, University Medical Center Mainz, Mainz, Germany.; 3 German Center for Cardiovascular Research (DZHK), Partner Site Rhine-Main, Mainz, Germany.; 4 Institute of Immunology, University Medical Center Mainz, Mainz, Germany.; 5 Cell Biology Unit, University Medical Center Mainz, Mainz, Germany and; 6 Departments of Biology and Physics, Wilkes University, Wilkes-Barre, Pennsylvania, USA.; 7 Institute for Molecular Medicine, University Medical Center Mainz, Mainz, Germany.; 8 Erich und Hanna Klessmann-Institut für Kardiovaskuläre Forschung und Entwicklung, Herz- und Diabeteszentrum NRW, Bad Oeynhausen, Germany.; 9 Helmholtz Institute for Translational Oncology (HI-TRON) Mainz, Germany and; 10 German Cancer Research Center (DKFZ), Heidelberg, Germany.; 11 Department of Immunology and Microbiology, Scripps Research, La Jolla, California, USA.

**Keywords:** Immunology, Cardiovascular disease, Coagulation, Heart failure

## Abstract

Despite major advances in acute interventions for myocardial infarction (MI), adverse cardiac remodeling and excess fibrosis after MI causing ischemic heart failure (IHF) remain a leading cause of death worldwide. Here we identify a profibrotic coagulation signaling pathway that can be targeted for improved cardiac function following MI with persistent ischemia. Quantitative phosphoproteomics of cardiac tissue revealed an upregulated mitogen-activated protein kinase (MAPK) pathway in human IHF. Intervention in this pathway with trametinib improves myocardial function and prevents fibrotic remodeling in a murine model of non-reperfused MI. MAPK activation in MI requires myeloid cell signaling of protease-activated receptor 2 linked to the cytoplasmic domain of the coagulation initiator tissue factor (TF). They act upstream of pro-oxidant NOX2 NADPH oxidase, ERK1/2 phosphorylation, and activation of profibrotic TGF-β1. Specific targeting with the TF inhibitor nematode anticoagulant protein c2 (NAPc2) starting 1 day after established experimental MI averts IHF. Increased TF cytoplasmic domain phosphorylation in circulating monocytes from patients with subacute MI identifies a potential thromboinflammatory biomarker reflective of increased risk for IHF and suitable for patient selection to receive targeted TF inhibition therapy.

## Graphical abstract

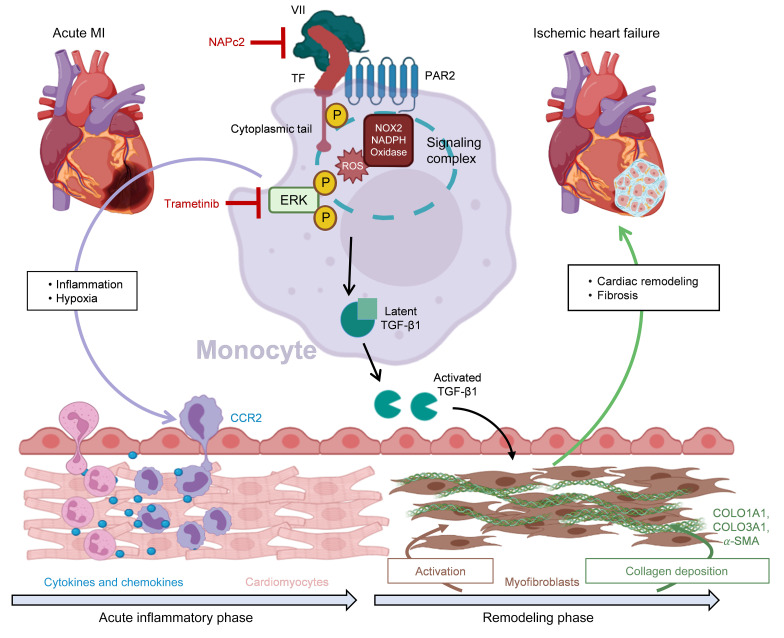

## Introduction

Proper wound healing depends on effective hemostasis, which allows regeneration and reconstitution of defective tissue function (1). Coronary thrombosis causing myocardial infarction (MI) (2) leads to irreversible tissue injury, particularly when timely reperfusion is not achieved. Activation of the innate and adaptive immune system mediates aspects of both cardiac dysfunction and functional repair (3). These immune responses support wound repair and collagen deposition that may lead to restoration of cardiac function or inflammation-mediated adverse left ventricular (LV) remodeling resulting in ischemic heart failure (IHF) (4). Targeting innate immune responses for secondary prevention after an MI was effective but did not result in a net clinical benefit owing to side effects (5), warranting tailored and more specific antiinflammatory approaches in coronary heart disease, in particular for the prevention of IHF.

Myeloid cells express tissue factor (TF), which forms a complex with factor VIIa (FVIIa) for FXa generation (6), and TF significantly contributes to multifaceted intravascular cell activation in immunothrombosis (7). Long-term anticoagulation is an established therapy to prevent major cardiovascular events (8, 9). Preclinical evidence suggests beneficial effects of anticoagulants (10) or blockade of protease-activated receptors (PARs) (11) in the infarcted myocardium. Inhibition of cardiomyocyte-specific TF or thrombin attenuates myocardial injury and inflammation (12), but the underlying mechanisms linking coagulation proteases to immune cell activation and outcomes after MI remain unclear.

The temporal transition of an inflammatory phase to fibrotic remodeling after MI depends on the release of growth factors like transforming growth factor-β1 (TGF-β1) (13) activating myofibroblasts (14). Coagulation proteases mediate clotting-independent PAR activation to regulate not only hematopoiesis (15) and tumor development (16) but also cardiac fibrosis in a model of heart failure with preserved ejection fraction by modulating TGF-β1 signaling (17). Transformation of tissue resident fibroblasts to activated myofibroblasts depends on TGF-β1 signaling (18), but cellular sources and mechanisms that regulate TGF-β1 production and activation in the infarcted myocardium remain incompletely understood.

In the present study, we identified persistent activation of coagulation as a prominent local feature in human IHF. We conducted genetic and pharmacological investigations to dissect crucial coagulation protease–mediated signaling events that promote excess cardiac fibrosis and adverse remodeling. These experiments identified an unexpected role for myeloid cell TF-PAR2 signaling as a crucial driver for TGF-β1 activation and a potential target for therapeutic intervention in MI to avert the development of IHF.

## Results

### Proteomic identification of coagulation and innate immune activation in human IHF.

**Figure 1:**
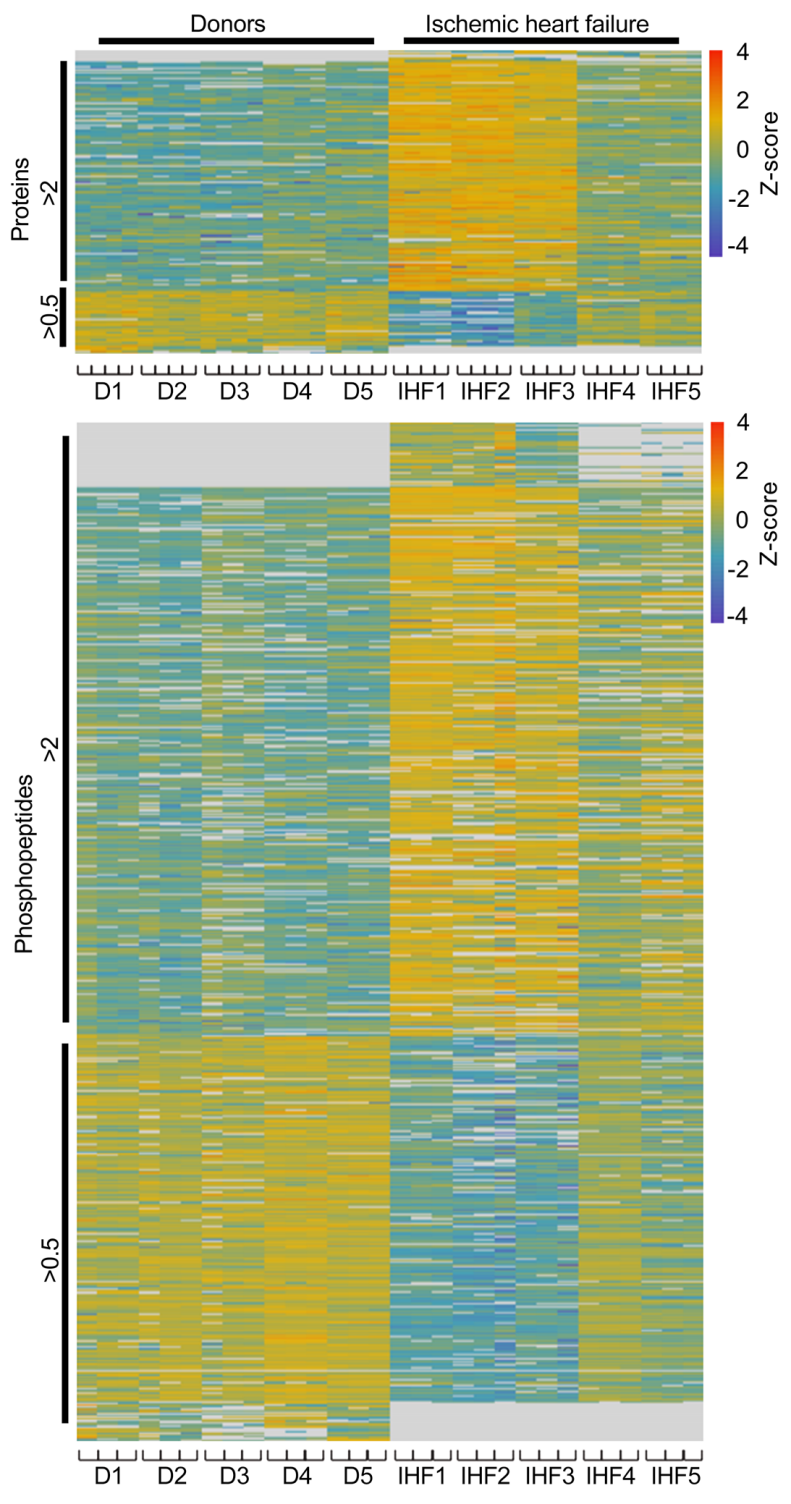
The proteomic signature of human IHF. Proteins isolated from cardiac tissue from human IHF (*n* = 5) and non-IHF donor hearts (controls, *n* = 5) were analyzed by label-free quantitative proteomics. Samples were measured in quadruplicate (proteins) and triplicate (phosphopeptides) liquid chromatography–tandem mass spectrometry (LC-MS/MS) runs. Heatmaps show the quantity profile for 208 proteins and 685 phosphopeptides significantly differing (*P* < 0.05, fold change > 2 or < 0.5, and identified in >60% of all LC-MS/MS replicates in either control or IHF group), log_10_-transformed and row-wise normalized using *z* score and sorted by descending fold change.

**Figure 2:**
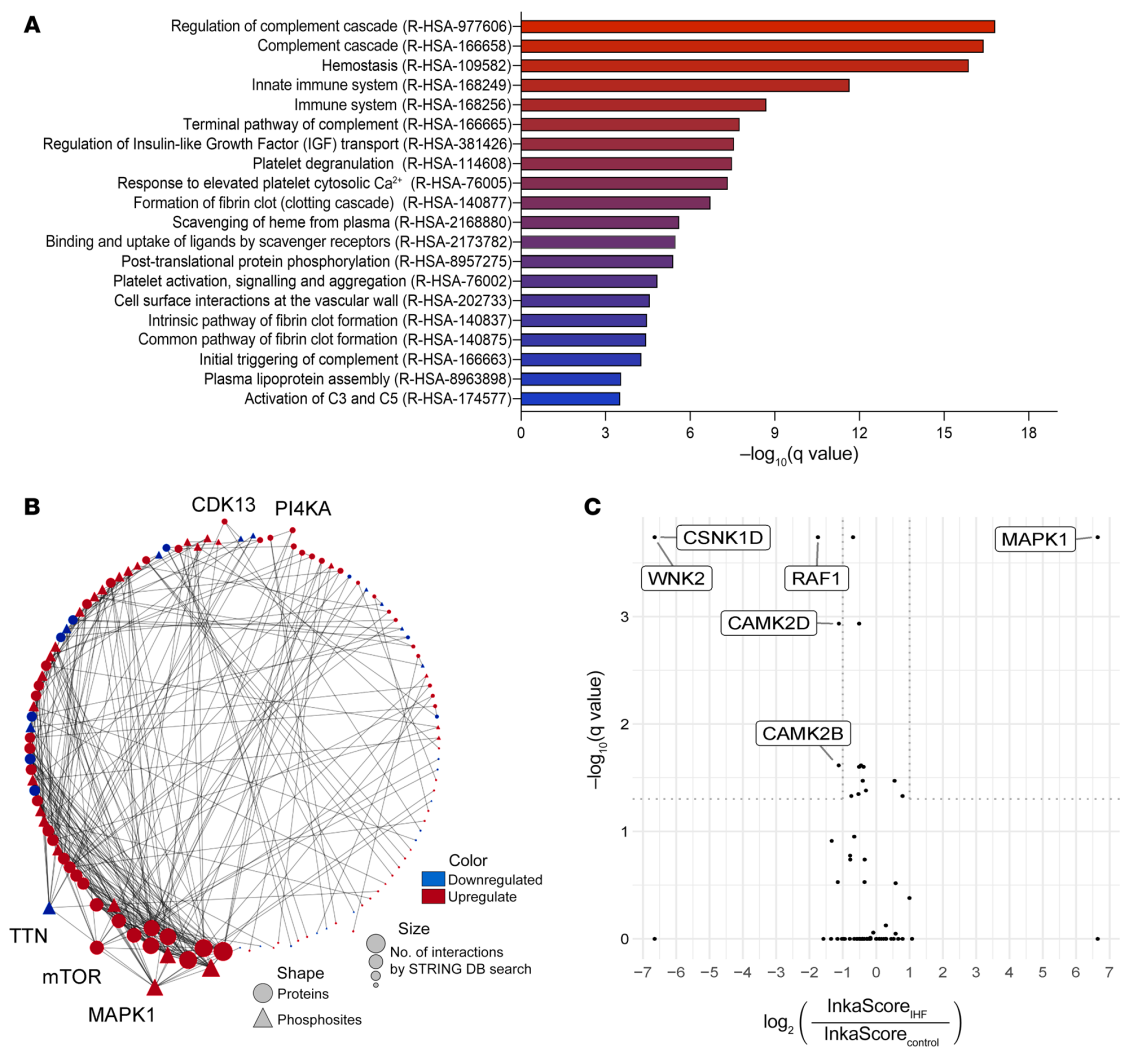
Enrichment of innate immune and coagulation pathways and of MAPK1 signaling in human IHF. (**A**) Reactome pathway enrichment analysis of differentially abundant proteins from Figure 1. (**B**) Differentially regulated (*P* < 0.05) nonphosphorylated and phosphorylated proteins were analyzed for their previously known protein-protein interactions between each other by Cytoscape STRING-DB search and sorted by the number of interactions in a circular layout. Among the present kinases, MAPK1 shows the most interactions, followed by mTOR, TTN, CDK13, PI4KA, and TAOK2. (**C**) Volcano plot depicting significantly differing Integrative Inferred Kinase Activity (InKA) scores between IHF and control groups.

To gain insight into the molecular pathophysiology of the failing human heart in chronic IHF, we performed an unbiased label-free quantitative proteomic and phosphoproteomic profiling of cardiac tissue obtained from 5 patients with severe advanced IHF compared with 5 control donor hearts. High-resolution-accuracy mass spectrometric analysis allowed for the quantification of 2,714 proteins and 10,601 phosphopeptides, of which 208 proteins and 685 phosphopeptides were significantly changed with fold changes greater than 2 or less than 0.5 in at least 60% of all measurements in 1 group (Figure 1, Supplemental Figures 1–6, and Supplemental Tables 1 and 2; supplemental material available online with this article; https://doi.org/10.1172/JCI156436DS1). Gene ontology analysis revealed a significant enrichment of pathways related to complement and coagulation cascades, innate immune pathways, platelet activation, and endocytosis in the IHF group relative to control (Figure 2A and Supplemental Table 3). Thrombosis and its sequelae are established hallmarks of acute MI and atherothrombotic complications in vascular diseases. The prominent changes in coagulation, complement, and innate immune responses suggested a novel and specific coagulation-inflammatory signature that persisted after acute ischemic events and might have ultimately led to chronic IHF in our samples of severe human ischemic heart disease.

Protein-protein interaction analysis with Cytoscape STRING-DB app identified phosphorylation of mitogen-activated protein kinase 1 (MAPK1, or extracellular signal–regulated kinase [ERK]) at Thr185 and/or Tyr187 as a central node in the integrated network kinase analysis (Figure 2B). In addition, Integrative Inferred Kinase Activity (InKA) analysis revealed that MAPK1 is a (hyper)active kinase (average InKA score of 49) in IHF but not in controls (Figure 2C). Thus, our analysis uncovered a central signaling pathway that may be related to the coagulation and innate immune system changes in human IHF. We therefore performed preclinical and clinical studies to establish the relevance of the MAPK pathway in MI and its progression to IHF as well as to elucidate the upstream coagulation-related signaling pathway involved and potential specific therapeutic targeting.

### Blockade of MAPK1 in a preclinical model of MI ameliorates the development of IHF.

**Figure 3:**
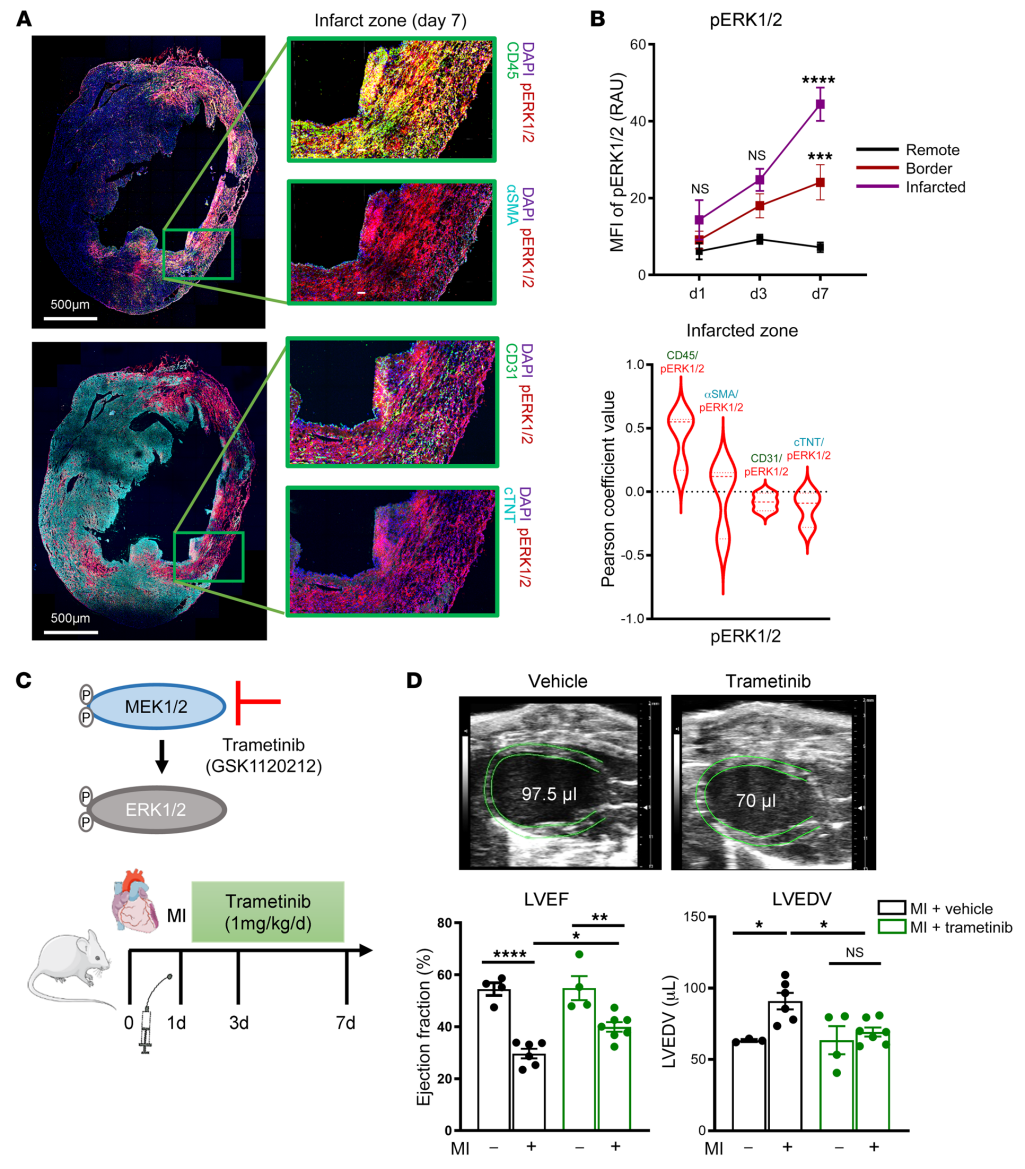
Infiltrating myeloid cells are the major source for increased MAPK1 activation in a preclinical model of non-reperfused MI. Confocal microscopy of whole LV myocardial cryosections obtained from *n* = 3–5 C57BL/6J mice after 7 days of permanent LAD ligation. (**A**) Representative images of p-ERK1/2^+^ cells costained for CD31, CD45, α-SMA, cTNT. Scale bars: 500 μm. (**B**) Top: Quantification of p-ERK1/2^+^ intensity in remote, border, and infarction regions at days 1, 3, and 7 after MI. Bottom: Quantification of colocalization analysis of p-ERK1/2 signal intensity with CD31, CD45, α-SMA, and cTNT in border and infarct regions at day 7 after MI using Pearson’s correlation coefficient. RAU, relative arbitrary units. (**C**) Experimental design: C57BL/6J mice were subjected to permanent LAD ligation versus sham surgery and given trametinib (1 mg/kg/d) or vehicle treatment once daily via oral gavage from day 1 to day 7. (**D**) High-frequency ultrasound echocardiography obtained in parasternal long axis with measurement of LV ejection fraction (LVEF, %) and LV end-diastolic volume (LVEDV, μL) on day 7 after operation. Ordinary 1-way ANOVA, Šidák’s multiple-comparison test; *n* = 4–6 animals per group. Data are shown as mean ± SEM. *P < 0.05, **P < 0.01, ***P < 0.001, ****P < 0.0001.

We used the well-established preclinical mouse model of MI induced by permanent ligation of the left anterior descending coronary artery (LAD) to study the development of IHF. We first localized phosphorylated ERK1/2 by immunofluorescence staining in the infarcted zone 7 days after MI (Figure 3A). ERK1/2 phosphorylation was detectable as early as 1 day after MI (Supplemental Figure 7) in the infarcted zone and increased from day 1 to day 7 in the infarcted and border zones (Figure 3B) but not in nonischemic remote regions of the infarcted hearts (Supplemental Figures 8 and 9). Colocalization of ERK1/2 phosphorylation with CD45^+^ immune cells, α-smooth muscle actin–positive (α-SMA^+^) myofibroblasts, CD31^+^ endothelial cells, and cTNT^+^ cardiomyocytes showed that ERK1/2 activation primarily occurred in infiltrating immune cells in the infarcted zone at day 7 (Figure 3B). Note that α-SMA did not colocalize with CD31 (Pearson’s coefficient value –0.2267), implying that α-SMA^+^ cells are primarily myofibroblasts and not smooth muscle cells in the ischemic myocardium. Immune cells, but not myofibroblasts, endothelial cells, or cardiomyocytes, were a prominent location for ERK1/2 activation throughout the course of acute MI in border and infarcted zones, but not remote and unaffected regions of the myocardium (Figure 3, A and B, and Supplemental Figures 7–9).

We then evaluated the functional contributions of the central MAPK hub identified by the (phospho)proteome analysis. We blocked the mitogen-activated extracellular signal–regulated kinase (MEK), an ERK1/2 activator, with the MEK1/2 inhibitor trametinib (Figure 3C). Long-term, high-dose trametinib regimens in cancer therapy have cardiotoxic side effects (19). Interestingly, short-term treatment with trametinib at a reduced dose of 1 mg/kg/d initiated 1 day and continued for 6 days after permanent LAD ligation attenuated the deterioration of cardiac function 7 days after MI (Figure 3D), while it did not significantly impact infarct size at days 1 and 3 after MI (Supplemental Figure 10A).

**Figure 4:**
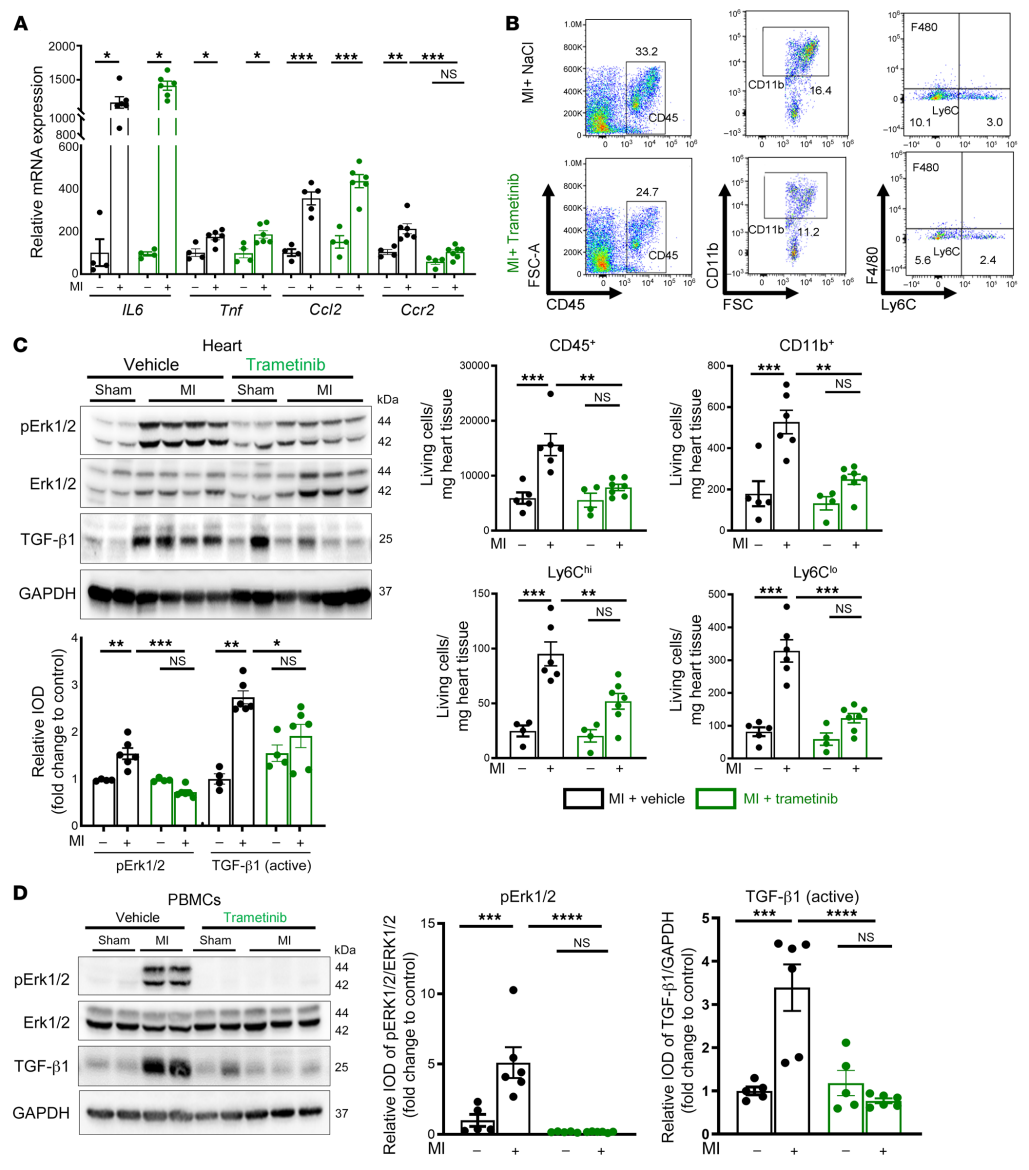
Inhibition of ERK1/2 activation attenuates myocardial remodeling and inflammation in permanent MI. Trametinib- and vehicle-treated mice were studied for 7 days after permanent LAD ligation. (**A**) Relative mRNA expression analysis of *Il6*, *Tnf*, *Ccl2*, and *Ccr2* from the infarcted myocardium. (**B**) Flow cytometry analysis of the infarcted myocardium obtained from vehicle- or trametinib-treated mice normalized to heart weight. Representative gating strategies for quantification of CD45^+^ leukocytes: CD45^+^CD90.2^–^B220^–^NK1.1^–^CD11b^+^ myelomonocytic cells, CD45^+^CD90.2^–^B220^–^NK1.1^–^CD11b^+^Ly6G^–^F4/80^–^Ly6C^hi^ monocytes, and CD45^+^CD90.2^–^B220^–^NK1.1^–^CD11b^+^Ly6G^–^F4/80^–^Ly6C^lo^ macrophages. (**C**) Protein expression analysis of p-ERK1/2 (normalized to total ERK1/2) and activated TGF-β1 (normalized to GAPDH) in infarcted myocardium obtained from vehicle- or trametinib-treated mice. IOD, integrated optical density. (**D**) Protein expression analysis of p-ERK1/2 (normalized to ERK1/2) and activated TGF-β1 (normalized to GAPDH) in PBMCs isolated from vehicle- or trametinib-treated mice. Ordinary 1-way ANOVA, Šidák’s multiple-comparison test; *n* = 4–6 animals per group. Data are shown as mean ± SEM. *P < 0.05, **P < 0.01, ***P < 0.001, ****P < 0.0001.

Cardiomyocyte death plays a crucial role in enhancing inflammatory responses in MI (20). Ly6G^+^ neutrophils and Ly6C^hi^ monocytes (3) and subsequent expansion of Ly6C^lo^ monocytes and macrophages (21) orchestrate the inflammatory reaction within the infarcted myocardium. Trametinib significantly reduced mRNA levels of monocytic C-C chemokine receptor 2 (*Ccr2*), which is essential for myeloid cell recruitment. Importantly, trametinib did not reduce mRNA levels of IL-6, TNF-α, and CCL (*Ccl2*), implying that the local release of inflammatory mediators after MI (4) was independent of the ERK1/2 pathway (Figure 4A). Cardiomyocyte apoptosis was marginally reduced and CD45^+^ immune cell infiltration was unchanged 1 and 3 days after MI (Supplemental Figure 10, B and C). In contrast, recruitment of CD45^+^CD11b^+^Ly6C^hi^ and Ly6C^lo^ myeloid cells into the infarcted heart was significantly reduced by trametinib at day 7 (Figure 4B and Supplemental Figure 11), in line with the reduced *Ccr2* mRNA levels. Trametinib therapy also had no significant effects on cardiac function in sham-operated mice from day 1 to day 7 and in ligated mice from day 1 to day 3 (Supplemental Figure 10D) and did not significantly interfere with p-JNK/SAK or p38 MAPK activation (Supplemental Figure 12A), demonstrating specificity.

TGF-β1 has been implicated in adverse LV remodeling and development of IHF (4, 22). In the infarcted zone, trametinib not only prevented phosphorylation of ERK, but also reduced activation of TGF-β1, the major driver of adverse cardiac fibrotic remodeling (Figure 4C). Likewise, mRNA levels of *COLO1A1* and *COLO3A1*, coding for collagen type I and III α1 chains, as well as *Posn* and *ACTA2*, coding for the fibrosis markers periostin and α-SMA, as potential targets of TGF-β1 signaling were significantly reduced in the infarcted myocardium (Supplemental Figure 12B). Next, we investigated the role of the MAPK pathway for TGF-β1 activation directly in immune cells. Protein expression analysis on isolated peripheral blood mononuclear cells (PBMCs) from the LAD-ligated mice revealed an increased ERK1/2 activation along with the increased TGF-β1 activation (Figure 4D). Thus, infiltrating CD45^+^ cells were the major source of ERK1/2 phosphorylation in the infarcted zone, and the MEK1/2 inhibitor trametinib prevented TGF-β1 activation in circulating PBMCs, suggesting that TGF-β1 activation was specifically regulated by immune cells after MI.

### Myeloid cell TF-PAR signaling is upstream of a profibrotic TGF-β1 pathway.

**Figure 5:**
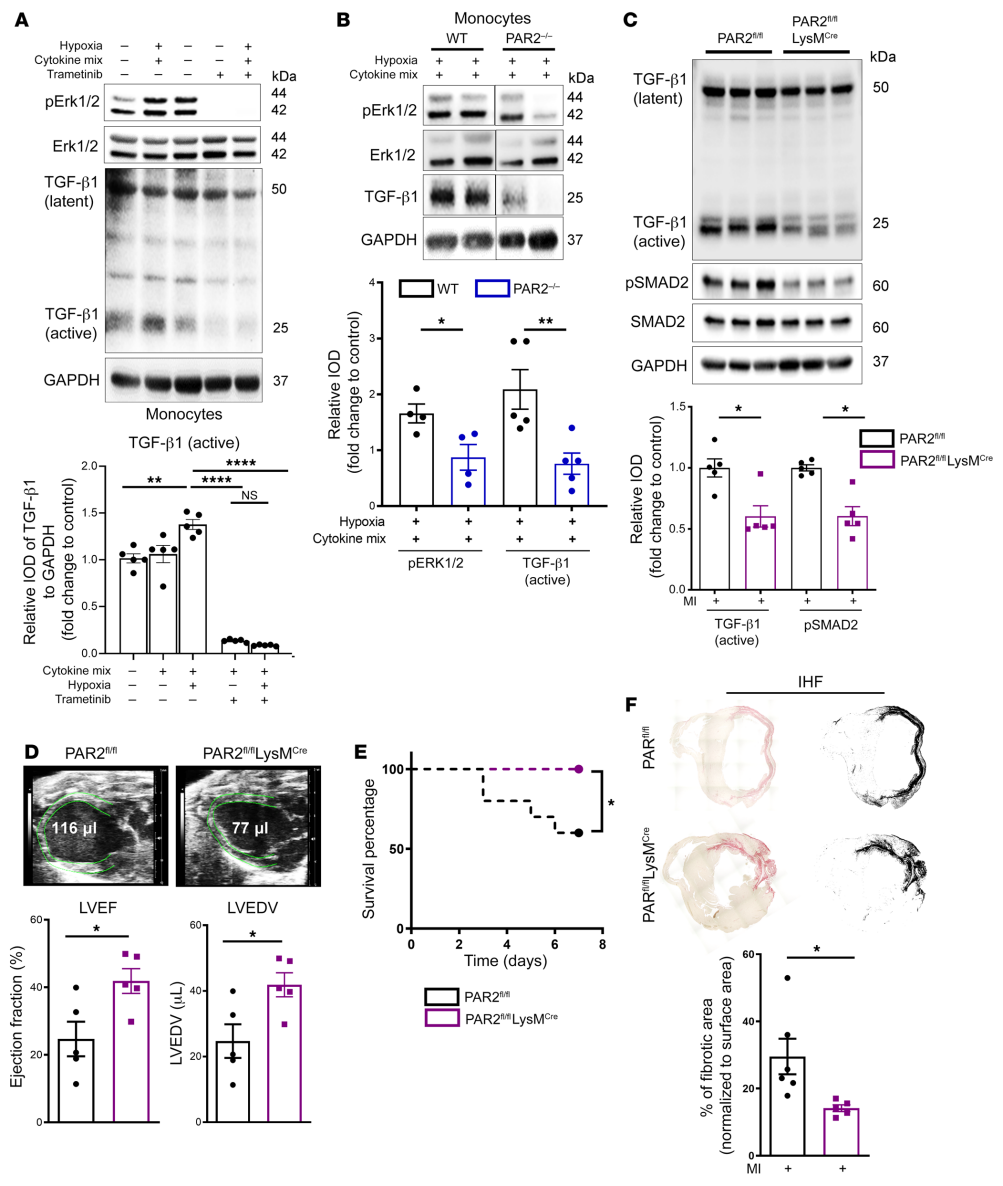
A profibrotic MEK1/2–TGF-β1 pathway is linked to PAR2-mediated ROS signaling in monocytes. (**A** and **B**) Protein expression analysis of monocytes isolated from WT mice and pretreated in vitro with trametinib (10 μM) for 1 hour (**A**), or isolated from PAR2^–/–^ versus WT mice (**B**). Cells were stimulated with an inflammatory cytokine cocktail containing IL-6, TNF-α, and CCL2 at a concentration of 20 ng/mL with and without hypoxia for 4 hours. Western blotting of p-ERK1/2 (normalized to total ERK1/2) and activated TGF-β1 (normalized to GAPDH). Ordinary 1-way ANOVA, Šidák’s multiple-comparison test; *n* = 5 replicates (2–3 mice were pooled for each sample). (**C**–**E**) PAR2^fl/fl^ and PAR2^fl/fl^ LysM^Cre^ littermates were subjected to permanent LAD ligation and investigated after 7 days; *n* = 5–10 animals per group. (**C**) Western blot analysis of activated TGF-β1 (normalized to GAPDH) and p-SMAD2 (normalized to total SMAD2) in the infarcted myocardium. Representative blots and quantification of biological replicates. (**D**) High-frequency ultrasound echocardiography obtained from PAR2^fl/fl^ LysM^Cre^ and PAR2^fl/fl^ littermate control mice with measurement of LVEF (%) and LVEDV (μL). Mann-Whitney test. (**E**) Kaplan-Meier survival analysis of permanently LAD-ligated PAR2^fl/fl^ LysM^Cre^ and PAR2^fl/fl^ littermate control mice over 7 days. Log-rank (Mantel-Cox) test. (**F**) Sirius red staining and deconvoluted images of fibrotic area on paraffin-embedded heart sections 4 weeks after permanent LAD ligation to induce IHF. Representative images and quantification of fibrotic areas normalized to surface area. Unpaired, 2-sided *t* test; *n* = 5 animals per group. Data are shown as mean ± SEM. *P < 0.05, **P < 0.01, ****P < 0.0001.

The proteome analysis of human ischemic myocardium demonstrated changes in complement and coagulation pathways in the context of an activated innate immune response, concomitant with ERK1/2 activation (Figure 2). Monocytes are known initiators of coagulation activation in a variety of pathological settings. We therefore next exposed isolated murine monocytes to both hypoxia and the inflammatory cytokines IL-6, TNF-α, and CCL2 (Figure 4A) detected in ischemic myocardium. While latent TGF-β1 expression was unchanged, monocytes exposed to both hypoxia and inflammatory cytokines significantly upregulated ERK1/2 phosphorylation and showed increased TGF-β1 activation, which was blocked by trametinib (Figure 5A). Complement activation influences the function of monocytic TF (23, 24) implicated in MI (25), oxidative stress, and thromboinflammation (6, 26). Western blot analysis of ischemic heart tissue confirmed that key coagulation components identified by the proteome screen were indeed upregulated in IHF patients relative to normal human donor hearts (Supplemental Figure 12C). TF in complex with its ligand FVIIa promotes sustained endosomal ERK1/2 signals by recruitment of the ERK1/2 scaffold β-arrestin to PAR2 and trafficking in complex with β_1_ integrin heterodimers (27). Implicating PAR2 as an upstream signal for monocyte ERK1/2 activation, we found that monocytes isolated from PAR2^–/–^ mice had reduced ERK1/2 phosphorylation and TGF-β1 activation when exposed to hypoxia plus cytokines (Figure 5B).

Based on these data, we subjected PAR2^fl/fl^ LysM^Cre^ mice to permanent LAD ligation for 7 days. Myeloid cell PAR2-deficient mice showed reduced TGF-β1 activation with unaltered latent TGF-β1 levels, reduced phosphorylation of the TGF-β1 target small mothers against decapentaplegic homolog 2 (SMAD2) in the myocardium (Figure 5C), and protection from cardiac dysfunction (Figure 5D). Immune cell recruitment into the infarcted myocardium was not diminished in myeloid cell PAR2-deficient mice (Supplemental Figure 12D). Four weeks after LAD ligation, PAR2^fl/fl^ LysM^Cre^ mice compared with PAR2^fl/fl^ littermate controls had significantly improved survival (Figure 5E) and improved cardiac function as well as decreased collagen deposition based on sirius red staining of myocardial cross sections (Figure 5F and Supplemental Figure 12E). Thus, myeloid cell PAR2 supports local TGF-β1 activation and drives cardiac remodeling following MI.

**Figure 6:**
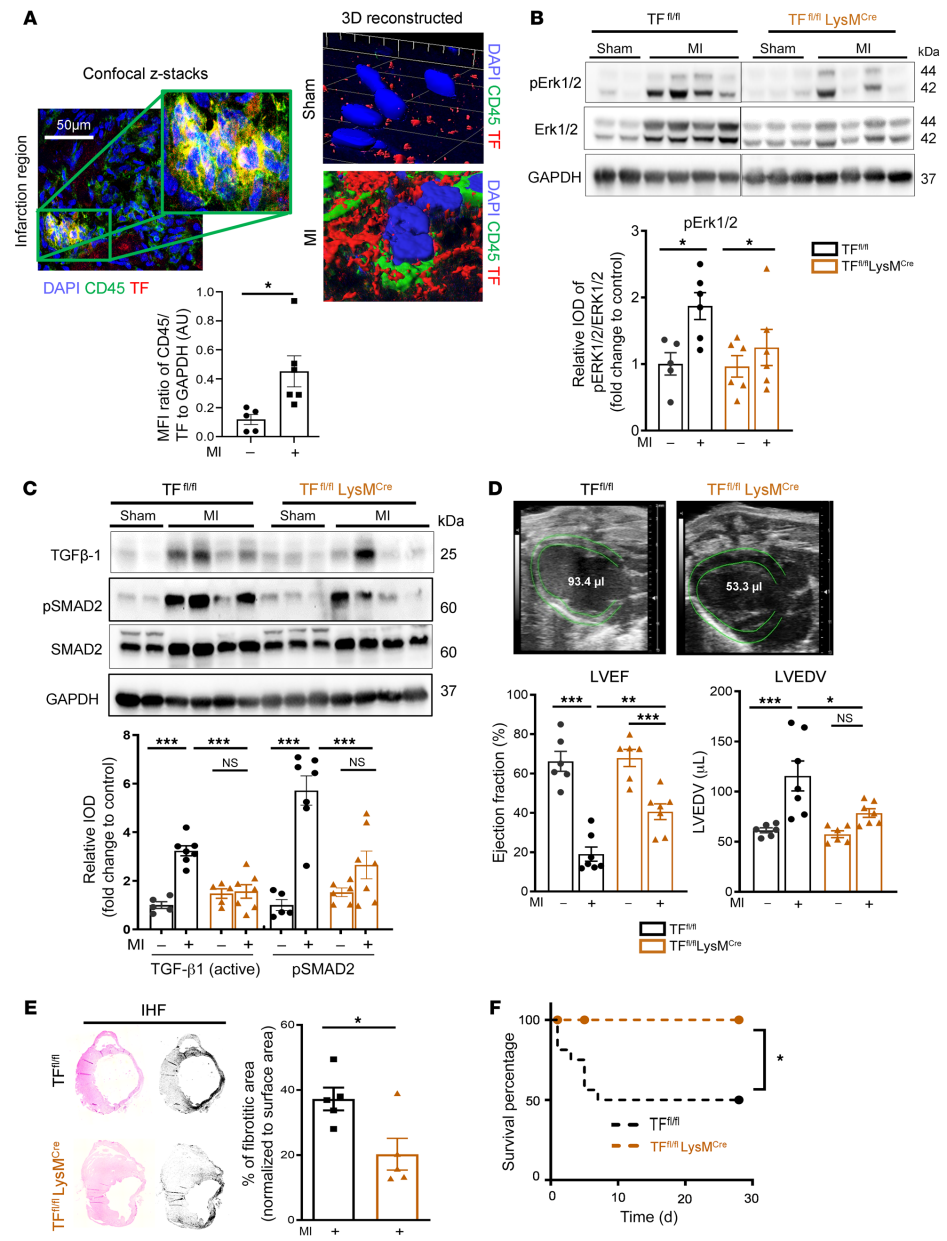
Myeloid cell–derived TF-PAR2 complex is required for TGF-β1 activation. (**A**) Confocal microscopy of myocardial cryosections obtained from *n* = 5 sham-operated and *n* = 5 LAD-ligated WT (C57BL/6J) mice at day 7. Representative images and quantification of TF^+^ cells costained for CD45. Unpaired, 2-sided *t* test. Scale bar: 50 μm. (**B**–**D**) TF^fl/fl^ LysM^Cre^ and TF^fl/fl^ littermates were subjected to permanent LAD ligation versus sham surgery and investigated after 7 days; *n* = 5–7 animals per group. (**B**) Protein expression analysis of p-ERK1/2 (normalized to total ERK1/2) in the infarcted myocardium. Ordinary 1-way ANOVA, Šidák’s multiple-comparison test. (**C**) Western blot analysis of activated TGF-β1 (normalized to GAPDH) and p-SMAD2 (normalized to total SMAD2) in the infarcted myocardium obtained from TF^fl/fl^ LysM^Cre^ and TF^fl/fl^ littermates. Representative blots and quantification of biological replicates. (**D**) High-frequency ultrasound echocardiography obtained from TF^fl/fl^ LysM^Cre^ and TF^fl/fl^ littermates. Ordinary 1-way ANOVA, Šidák’s multiple-comparison test. (**E**) Sirius red staining and deconvoluted images of fibrotic area on paraffin-embedded heart sections 4 weeks after permanent LAD ligation to induce IHF versus sham surgery. Representative images and quantification of fibrotic areas normalized to surface area. Ordinary 1-way ANOVA, Šidák’s multiple-comparison test; *n* = 5 animals per group. (**F**) Kaplan-Meier survival analysis of permanently LAD-ligated TF^fl/fl^ LysM^Cre^ and TF^fl/fl^ littermate mice over 4 weeks. Log-rank (Mantel-Cox) test; *n* = 10–15 animals per group. Data are shown as mean ± SEM. *P < 0.05, **P < 0.01, ***P < 0.001.

Costaining for TF and CD45 revealed a significant increase of CD45/TF double-positive cells in the infarcted myocardium (Figure 6A). Confocal microscopy imaging of infarcted myocardium of TF^fl/fl^ LysM^Cre^ mice confirmed TF localization in myeloid cells (Supplemental Figure 13A). TGF-β1–mediated SMAD2 activation in the infarcted myocardium is known to start at day 7 and persist until 28 days after MI (22, 28). There was no difference in infarct size and cardiomyocyte apoptosis 1 day after MI in TF^fl/fl^ LysM^Cre^ mice relative to TF^fl/fl^ littermate controls (Supplemental Figure 13, B and C). In line with myeloid cell PAR2 deletion, TF deficiency led to significantly reduced cardiac ERK1/2 activation (Figure 6B), TGF-β1 activation, and SMAD2 phosphorylation (Figure 6C) as well as mRNA expression of TGF-β1–induced *COLO1A1*, *COLO3A1*, and *ACTA2* (Supplemental Figure 14A). This resulted in improved cardiac function (Figure 6D) without influencing myeloid cell infiltration into the infarcted myocardium 7 days after MI (Supplemental Figure 14B).

**Figure 7:**
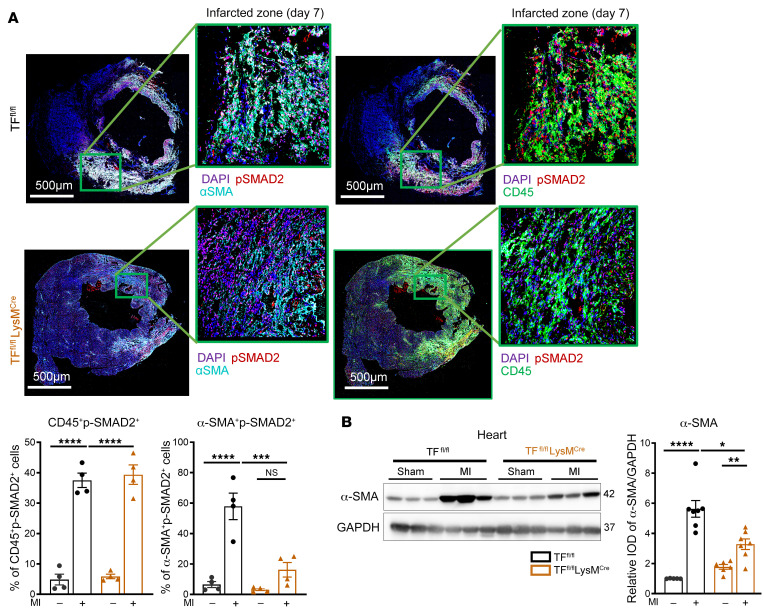
Myeloid cell to myofibroblast crosstalk for SMAD2 phosphorylation. (**A**) Confocal microscopy analysis of CD45^+^p-SMAD2^+^ and α-SMA^+^p-SMAD2^+^ cells in the infarcted myocardium compared with remote myocardium obtained from TF^fl/fl^ LysM^Cre^ and TF^fl/fl^ littermates at day 7 after MI. Scale bars: 500 μm. (**B**) Protein expression analysis of α-SMA (normalized to GAPDH) in the infarcted myocardium compared with myocardium of sham-operated TF^fl/fl^ LysM^Cre^ mice at day 7 after MI. Ordinary 1-way ANOVA, Šidák’s multiple-comparison test; *n* = 4–7 animals per group. Data are shown as mean ± SEM. *P < 0.05, **P < 0.01, ***P < 0.001, ****P < 0.0001.

Long-term follow up of TF^fl/fl^ LysM^Cre^ mice revealed reduced collagen deposition, improved survival (Figure 6, E and F), and better cardiac function 4 weeks after MI in comparison with TF^fl/fl^ controls (Supplemental Figure 14C). Immunofluorescence staining revealed an increase of both CD45^+^p-SMAD2^+^ and α-SMA^+^p-SMAD2^+^ cells in the infarcted myocardium 7 days after MI (Figure 7A). Myeloid cell TF deletion did not reduce CD45^+^p-SMAD2^+^ cells, but α-SMA^+^p-SMAD2^+^ cells as well as total α-SMA protein expression in the myocardium were significantly reduced in TF^fl/fl^ LysM^Cre^ mice compared with TF^fl/fl^ controls (Figure 7, A and B). Thus, TF-PAR2 signaling in infiltrating myeloid cells is responsible for hyperactivation of the MAPK pathway, TGF-β1 activation, and activated myofibroblast accumulation leading to fibrotic cardiac remodeling after MI and the development of IHF.

### TF cytoplasmic domain signaling is linked to NOX2/ERK-dependent TGF-β1 activation in permanent MI.

We next asked whether TF makes direct signaling contributions to profibrotic TGF-β1 activation. Ligation of TF by FVIIa activates rac and p38 dependent on the TF cytoplasmic domain (29). In the context of PAR signaling, the TF cytoplasmic tail binds the regulatory subunit of PI3 kinase and rac adaptor p85 (27) and recruits the NADPH oxidase for endosomal translocation and reactive oxygen species (ROS), primarily superoxide anion (O_2_^•−^), production (26). We found that TF^fl/fl^ LysM^Cre^ mice with permanent LAD ligation had significantly reduced cardiac NOX2 expression compared with controls and that monocytes isolated from PAR2^–/–^ mice had reduced NOX2 expression when exposed to hypoxia plus cytokines (Supplemental Figure 15, A and B). Likewise, O_2_^•−^ formation in the ischemic myocardium of PAR^fl/fl^ LysM^Cre^ mice was reduced in comparison with PAR^fl/fl^ littermate controls (Supplemental Figure 15C).

**Figure 8:**
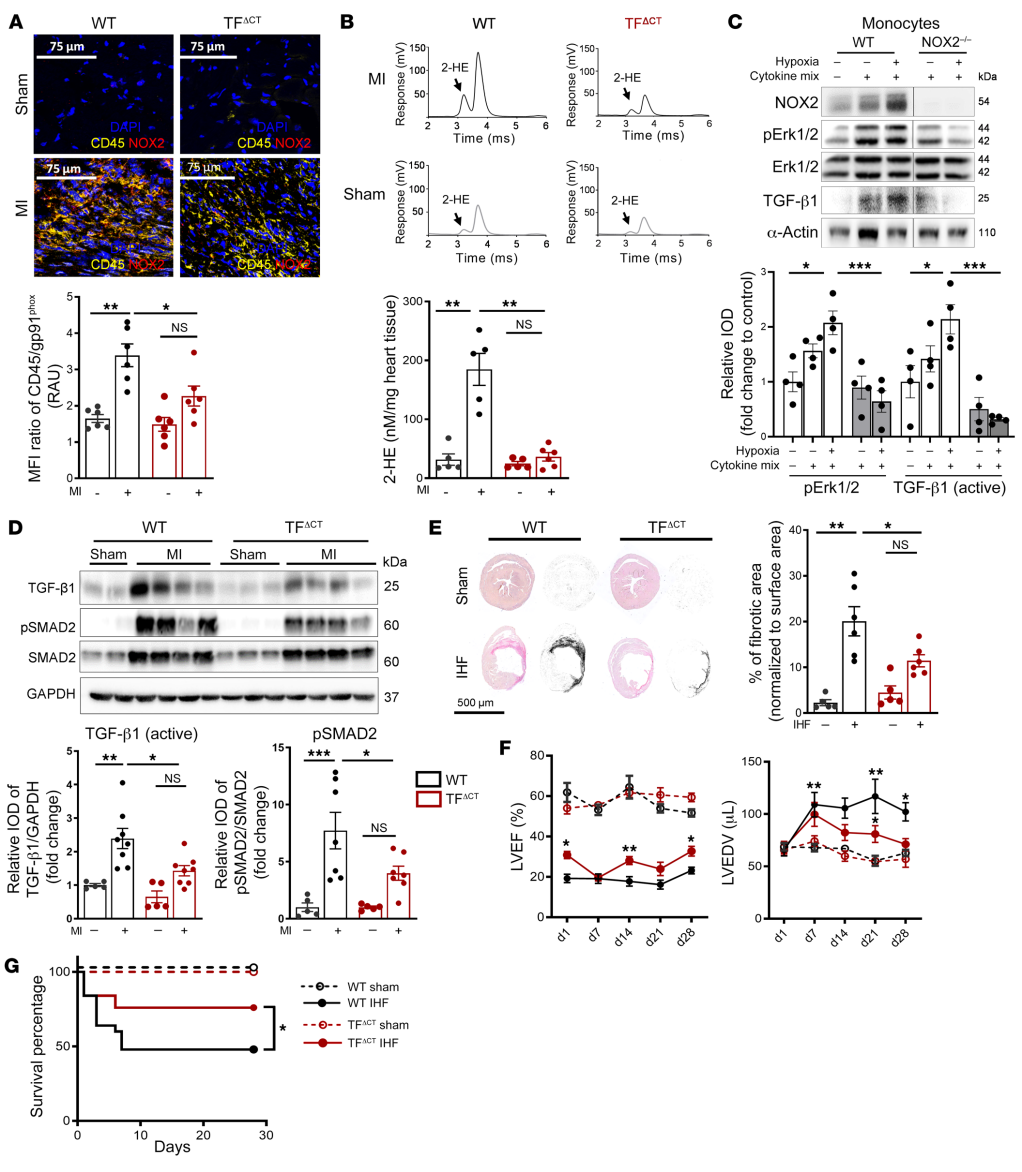
TF cytoplasmic tail deletion attenuates ROS production and ERK1/2–TGF-β1 signaling–dependent cardiac fibrosis and improves cardiac function. WT and TF^ΔCT^ mice were subjected to permanent LAD ligation versus sham surgery and investigated after 7 days and 4 weeks. (**A**) Confocal microscopy of myocardial cryosections. Representative images and quantification of MFI of CD45/gp91^phox^ double-positive cells. Scale bars: 75 μm. (**B**) Assessment of superoxide formation in infarcted myocardium by dihydroethidium-HPLC analysis. Representative chromatogram of 2-hydroxyethidium (2-HE), the oxidation product of DHE, and quantification normalized to weight of the infarcted tissue. Ordinary 1-way ANOVA, Šidák’s multiple-comparison test; *n* = 5–6 animals per group. (**C**) Protein expression analysis of monocytes isolated from NOX2^–/–^ animals and stimulated with an inflammatory cytokine cocktail containing IL-6, TNF-α, and CCL2 at a concentration of 20 ng/mL with and without hypoxia for 4 hours. Western blotting of p-ERK1/2 (normalized to ERK1/2) and activated TGF-β1 (normalized to GAPDH). Ordinary 1-way ANOVA, Šidák’s multiple-comparison test; *n* = 4 replicates per group (2–3 mice were pooled for each sample). (**D**) Western blot analysis of activated TGF-β1 (normalized to GAPDH) and p-SMAD2 (normalized to total SMAD2) in infarcted myocardium obtained from WT or TF^ΔCT^ mice after 7 days of MI. Representative blots and quantification of biological replicates. Ordinary 1-way ANOVA, Šidák’s multiple-comparison test; *n* = 5–7 animals per group. (**E**) Sirius red staining and deconvoluted images of fibrotic area on paraffin-embedded heart sections 4 weeks after permanent LAD ligation to induce IHF versus sham surgery. Representative images and quantification of fibrotic areas normalized to surface area. Ordinary 1-way ANOVA, Šidák’s multiple-comparison test; *n* = 5–7 animals per group. (**F**) Longitudinal echocardiographic studies over 4 weeks for LVEF (%) and LVEDV (μL) in parasternal long axis M-mode. Two-way ANOVA, Bonferroni’s multiple-comparisons test; *n* = 6–17 animals per group. (**G**) Kaplan-Meier survival analysis of permanently LAD-ligated versus sham-operated C57BL/6J and TF^ΔCT^ mice over 4 weeks. Log-rank (Mantel-Cox) test; *n* = 15–20 animals per group. Data are shown as mean ± SEM. *P < 0.05, **P < 0.01, ***P < 0.001.

In line with results in TF^fl/fl^ LysM^Cre^ and PAR^fl/fl^ LysM^Cre^ mice, cytoplasmic tail–deficient mice (TF^ΔCT^ mice) had markedly reduced CD45^+^NOX2^+^ immune cell infiltration (Figure 8A) and O_2_^•−^ formation (Figure 8B) in the infarcted myocardium. In addition, circulating mononuclear cells isolated from TF^ΔCT^ mice after MI had significantly decreased expression of NOX2 and regulatory subunit p67^phox^ (Supplemental Figure 15D) in comparison with WT mice 7 days after permanent LAD ligation. TGF-β1 and ROS can act as a feed-forward mechanism for fibrosis (30), and NOX2 significantly contributes to oxidative stress and cardiac remodeling after MI (31). Importantly, NOX2^–/–^ monocytes exposed to cytokine mix and hypoxia had significantly decreased ERK1/2 phosphorylation and TGF-β1 activation, indicating a central role for NADPH oxidase–derived ROS (Figure 8C). In line with the reduced O_2_^•−^ production, infarcted myocardium of TF^ΔCT^ mice relative to WT showed markedly reduced ERK1/2 phosphorylation, decreased TGF-β1 activation, and reduced TGF-β1 signaling, based on phosphorylation of SMAD2 and profibrotic α-SMA induction (Figure 8D and Supplemental Figure 15E). Importantly, the phosphorylation of the alternative TF signaling target p38 MAPK (29) (Supplemental Figure 15F) was not altered in TF^ΔCT^ mice, underscoring the specificity of the ERK pathway in cardiac remodeling after MI.

Deletion of TF’s cytoplasmic tail had no effect on infarct size and cardiomyocyte apoptosis 1 day after MI, based on Masson’s trichrome and TUNEL staining (Supplemental Figure 15, G and H). Because early TGF-β1 signaling is required for late cardiac remodeling in the development of IHF after MI (22, 28), we examined TF^ΔCT^ mice 4 weeks after MI. Sirius red staining revealed increased collagen deposition and larger fibrotic areas in the hearts of LAD-ligated compared with sham-operated mice, which were significantly reduced in TF^ΔCT^ mice (Figure 8E). These morphological improvements were associated with protection from functional deterioration in TF^ΔCT^ mice with significantly less dilated and better-contracting left ventricles (Figure 8F) and improved survival in comparison with control mice (Figure 8G).

### TF cytoplasmic domain phosphorylation in experimental MI and in the clinical settings of IHF.

**Figure 9:**
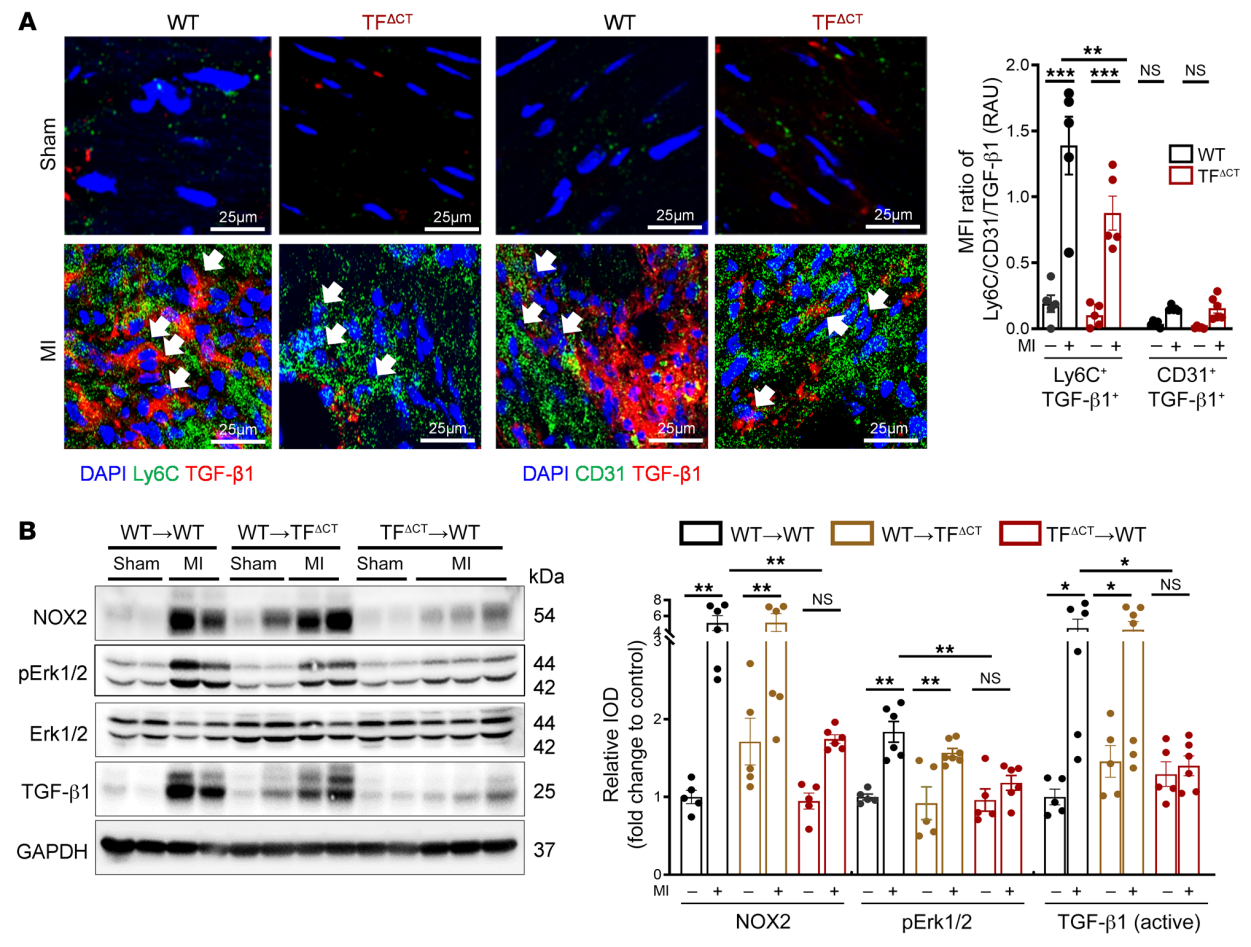
Myeloid cell TF cytoplasmic domain phosphorylation mediates ERK1/2–TGF-β1–dependent cardiac remodeling in permanent LAD ligation. (**A**) Confocal microscopy of myocardial cryosections obtained from WT (C57BL/6J) and TF^ΔCT^ mice. Representative images and quantification of MFI of Ly6C^+^TGFβ-1^+^ and CD31^+^TGF-β1^+^ cells. Ordinary 1-way ANOVA, Šidák’s multiple-comparison test; *n* = 5 animals per group. Scale bars: 25 μm. (**B**) Mice with transplanted BM were subjected to permanent LAD ligation versus sham surgery and investigated 7 days later. Western blot analysis of NOX2 (normalized to GAPDH), p-ERK1/2 (normalized to total ERK1/2), and TGF-β1 (normalized to GAPDH) in infarcted myocardium obtained from chimeric mice. Ordinary 1-way ANOVA, Šidák’s multiple-comparison test; *n* = 5–7 animals per group. Data are shown as mean ± SEM. *P < 0.05, **P < 0.01, ***P < 0.001.

Immunohistochemical staining of ischemic myocardium showed a relative abundance of TGF-β1^+^Ly6C^+^ inflammatory cells compared with TGF-β1^+^CD31^+^ cells in control mice, but not in TF^ΔCT^ mice (Figure 9A). To further analyze the role of the TF cytoplasmic tail in myeloid cell TGF-β1 activation, we generated bone marrow (BM) chimeras of TF^ΔCT^ and WT mice. After 9–10 weeks of confirmed engraftment (Supplemental Figure 16, A and B), permanent MI was induced in chimeric mice for analysis 7 days later. Whereas CD45^+^CD11b^+^ myeloid cell recruitment to the infarcted myocardium was indistinguishable between transplant groups (Supplemental Figure 16C), only chimeras with BM from TF^ΔCT^ mice (TF^ΔCT^→WT) had reduced cardiac NOX2 expression, ERK1/2 phosphorylation, and TGF-β1 activation (Figure 9B) paralleled by attenuated SMAD2 phosphorylation after MI (Supplemental Figure 17A). Six weeks after permanent LAD ligation, only TF^ΔCT^→WT mice showed improved cardiac function, thereby phenocopying the TF^ΔCT^ animals (Supplemental Figure 17B). Thus, this preclinical evidence links myeloid cell TF-PAR2 signaling to TF cytoplasmic tail–dependent NOX2 activation, ERK phosphorylation, and TGF-β1 activation in MI and adverse cardiac remodeling leading to IHF.

**Figure 10:**
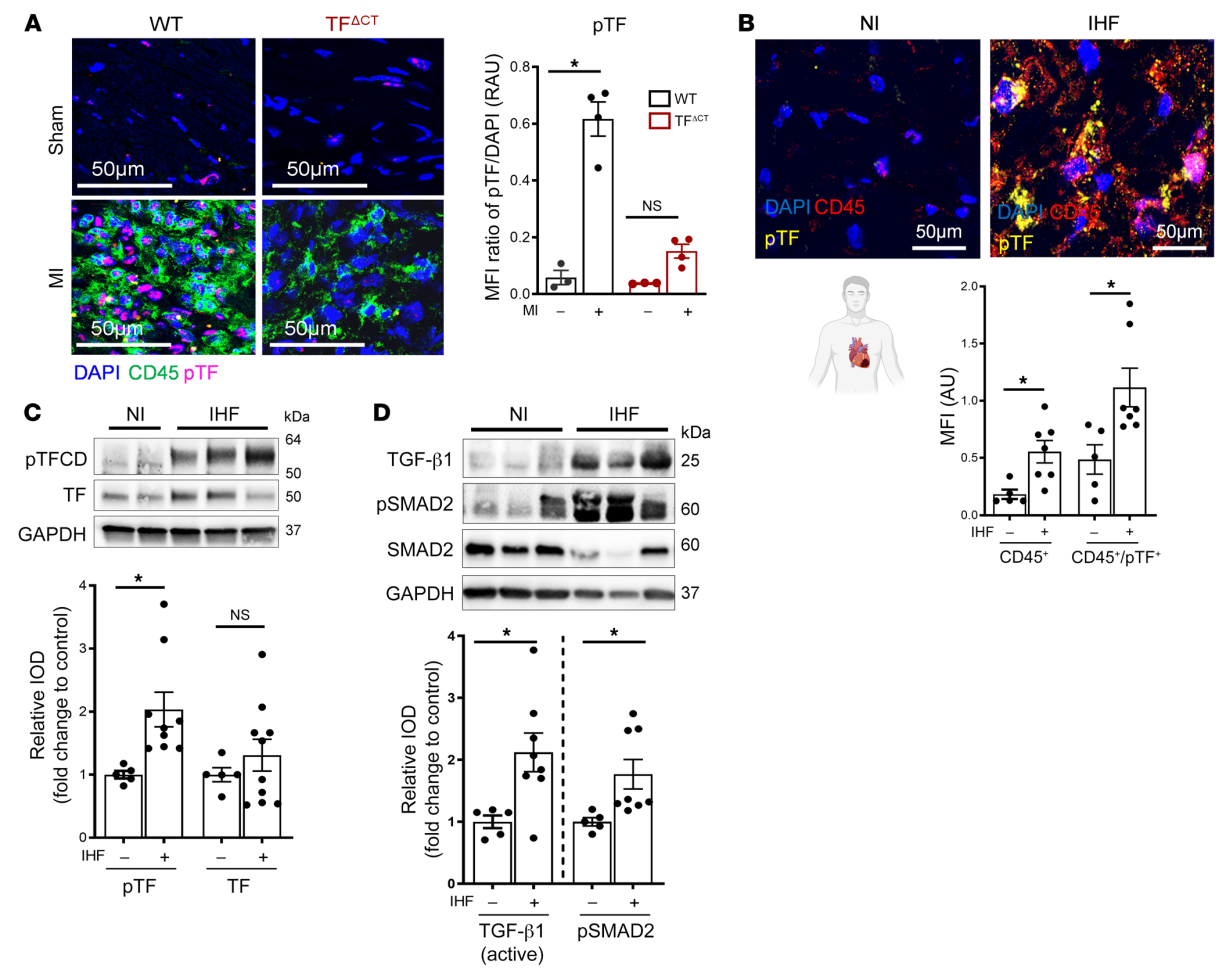
TF cytoplasmic domain phosphorylation–dependent increased TGF-β1 activation in clinical setting of MI. (**A**) Representative confocal images of phosphorylation status of TF in infarcted myocardium obtained from WT or TF^ΔCT^ mice after 7 days. Representative images and quantification of biological replicates. Kruskal-Wallis test and Dunn’s multiple-comparison test; *n* = 3–4 animals per group. Scale bars: 50 μm. (**B**) Representative immunofluorescence confocal microscopy images of CD45^+^ and CD45/p-TF double-positive cells in human myocardium specimens obtained from *n* = 5 nonischemic (NI) donor hearts and *n* = 7 IHF patients. Quantification of biological replicates. Mann-Whitney test. Scale bars: 50 μm. (**C** and **D**) Western blot analysis and quantification of human LV tissue obtained from *n* = 5 nonischemic donor hearts and *n* = 9 IHF patients for p-TF (normalized to total TF) and TF (**C**) or TGF-β1 (normalized to GAPDH) and p-SMAD2 (normalized to total SMAD2) (**D**). Mann-Whitney test. Data are shown as mean ± SEM. *P < 0.05.

Phosphorylation of the TF cytoplasmic domain was detectable specifically in CD45^+^ cells 7 days after experimental MI in WT but not in TF^ΔCT^ mice (Figure 10A), suggesting that activation of the TF-dependent profibrotic pathway can be identified by measurement of this posttranslational modification of TF. We employed previously validated phosphorylation-specific antibodies against the TF cytoplasmic domain (32) to translate this finding to human IHF. The numbers of CD45^+^ cells stained for phosphorylated TF were markedly increased in LV tissue samples obtained from IHF patients as compared with donor heart tissue (Figure 10, B and C, and Supplemental Table 4). This increased TF phosphorylation was accompanied by upregulation of IL-6, CCL2, and CCR2 in the heart tissue (Supplemental Figure 17C), indicating myeloid cell recruitment, as well as increased TGF-β1 activation and downstream phosphorylation of SMAD2 (Figure 10D), indicating subsequent cardiac fibrotic remodeling.

**Figure 11:**
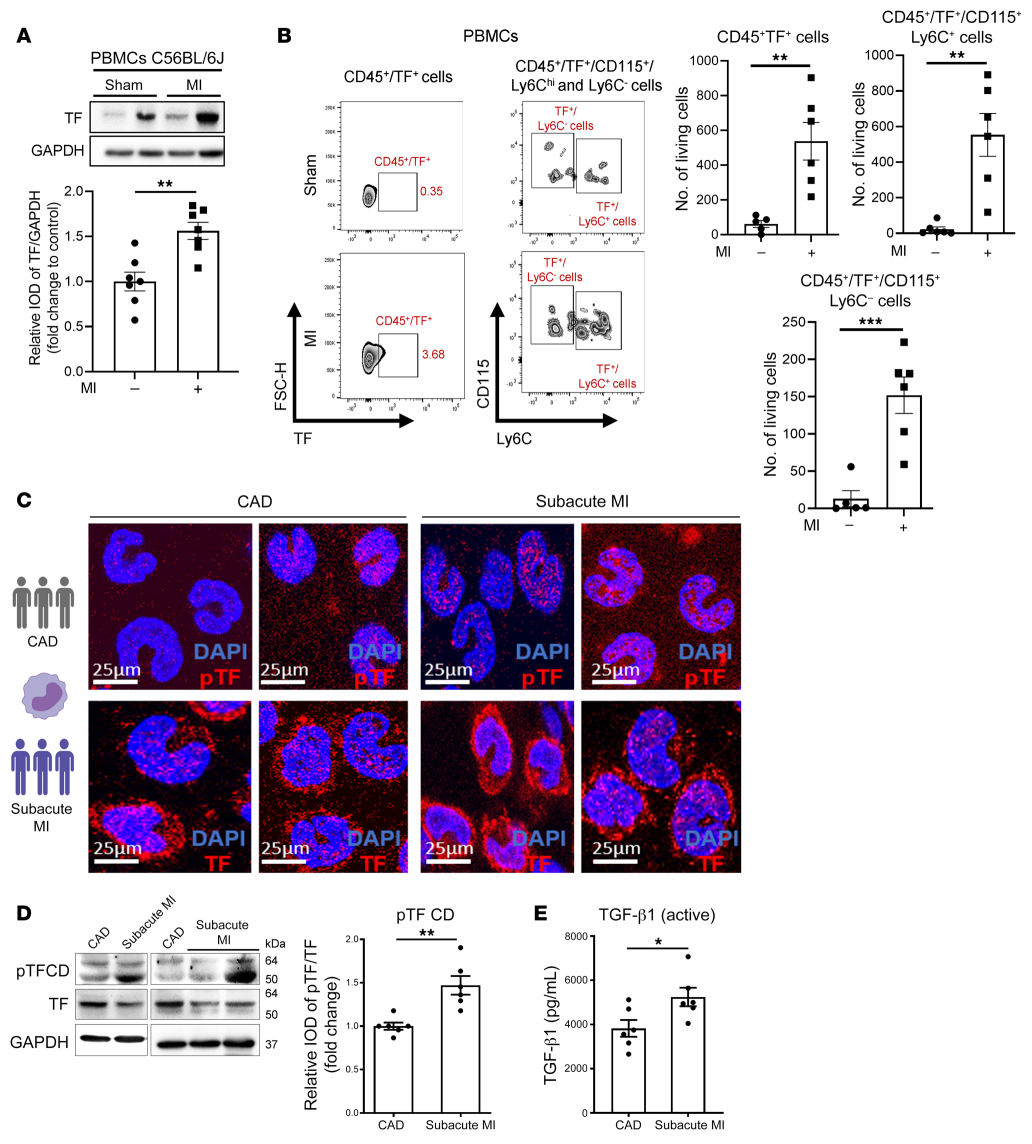
Phosphorylation of TF cytoplasmic domain on circulating monocytes in ongoing cardiac ischemic damage. Circulating PBMCs were isolated from C57BL/6J mice at day 7 after permanent LAD ligation. (**A**) Western blot analysis of TF expressed by PBMCs. Unpaired, 2-sided *t* test; *n* = 5–7 animals per group. (**B**) Flow cytometry analysis of the PBMCs. Representative counter-plots and quantification of CD45^+^TF^+^, CD45^+^TF^+^CD115^+^Ly6C^+^, and TF^+^CD115^+^Ly6C^–^ monocytes per 1 mL whole blood. Mann-Whitney 2-sided *t* test; *n* = 5–6 animals per group. (**C**–**E**) Representative confocal microscopy of isolated monocytes stained for p-TF (red), TF (red), and DAPI (blue) (**C**) followed by Western blot analysis of monocytic TF cytoplasmic domain phosphorylation (4G6) and TF (10H10) (**D**) and plasma levels of activated TGF-β1 (**E**) in samples obtained from patients with subacute MI (*n* = 6) and stable coronary artery disease (CAD) (*n* = 6) as described in Supplemental Table 5. Mann-Whitney unpaired, 2-sided *t* test. Scale bars: 25 μm. Data are shown as mean ± SEM. *P < 0.05, **P < 0.01, ***P < 0.001.

TF was upregulated on circulating PBMCs in mice after MI (Figure 11, A and B). We next asked whether TF phosphorylation in liquid biopsies could be used for identifying patients at risk for IHF. Patients with subacute, prolonged MI that is not timely reperfused have a 2-fold higher risk of death and of developing heart failure compared with MI patients presenting early for reperfusion therapy (33). In a sample taken from our observational MICAT study, we focused on patients with subacute MI compared with stable coronary artery disease (CAD) admitted for percutaneous coronary intervention (PCI) (Supplemental Table 5). TF cytoplasmic domain phosphorylation in circulating monocytes and plasma levels of active TGF-β1 were significantly increased in subacute MI compared with stable CAD patients (Figure 11, C–E). These data indicated that myeloid cell TF phosphorylation could serve as a marker to identify patients at increased risk of developing IHF and adverse remodeling following MI with ongoing cardiac ischemia.

### Pharmacological targeting of TF-FVIIa improves cardiac function by preventing TGF-β1 activation.

**Figure 12:**
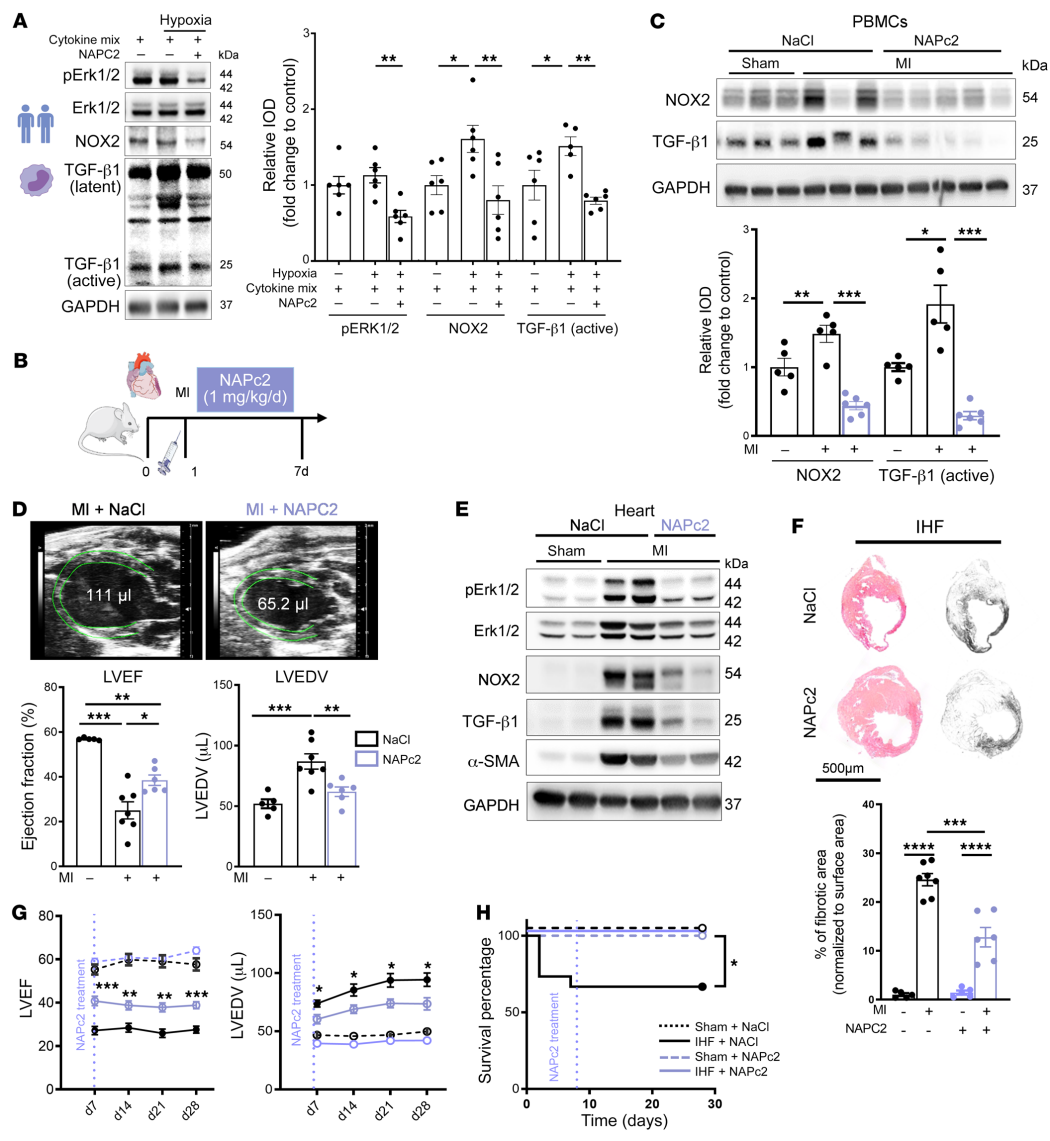
Pharmacological targeting of TF-FVIIa improves cardiac function by preventing TGF-β1 activation. (**A**) Protein expression analysis of p-ERK1/2 (normalized to total ERK1/2), NOX2, and TGF-β1 (normalized to GAPDH) on isolated human monocytes exposed to hypoxia in the presence of cytokine cocktail mix (20 ng/mL) with and without NAPc2 (200 ng/mL). Representative blots and quantification; *n* = 6 healthy individuals per group. (**B**) Experimental design: Mice were injected with NAPc2 (1 mg/kg/d) versus sham once daily by i.p. injection from day 1 through day 7. (**C**) Western blot analysis of NOX2 and TGF-β1 (normalized to GAPDH) obtained from PBMCs of the experimental animals 7 days after MI. Representative images and quantification of replicates. (**D** and **E**) High-frequency echocardiography obtained in parasternal long axis (PLAX) with measurement of LVEF and LVEDV on day 7 after LAD ligation (**D**) and representative blots for protein expression analysis of p-ERK1/2, NOX2, TGF-β1, and α-SMA (**E**) in infarcted myocardium obtained from vehicle- or NAPc2-treated mice. Ordinary 1-way ANOVA, Šidák’s multiple-comparison test; *n*= 5–7 animals per group. (**F**–**H**) Mice were injected with NAPc2 (1 mg/kg/d) or vehicle (1 mg/kg/d) once daily by i.p. injection from day 1 through day 7 followed by longitudinal analysis. (**F**) Representative images and quantification of fibrotic areas normalized to surface area. Ordinary 1-way ANOVA, Šidák’s multiple-comparison test; *n* = 5–7 animals per group. (**G**) Echocardiographic studies over 4 weeks for LVEF (%) and LVEDV (μL) in PLAX M-mode. (**H**) Kaplan-Meier survival analysis of LAD-ligated versus sham-operated NAPc2- and vehicle-treated mice after 4 weeks. Two-way ANOVA, Bonferroni’s multiple-comparison test, log-rank (Mantel-Cox) test; *n* = 6–15 animals per group. Data are shown as mean ± SEM. *P < 0.05, **P < 0.01, ***P < 0.001, ****P < 0.0001.

We next targeted TF signaling pharmacologically after MI. Nematode anticoagulant protein C2 (NAPc2) blocks TF signaling and coagulation by forming an inhibited TF-FVIIa-FX(a) complex (34). NAPc2 has been tested in PCI without increasing bleeding risk in combination with antiplatelet and standard heparin therapy (35) and was recently evaluated in a clinical trial in COVID-19 (36) without major safety concerns. Recapitulating the findings in mouse monocytes, isolated human monocytes exposed to inflammatory cytokines and hypoxia had increased NOX2 expression and TGF-β1 activation, which were suppressed by NAPc2 along with ERK1/2 phosphorylation (Figure 12A).

In our preclinical model 7 days after MI, circulating myeloid cells had increased levels of NOX2 and active TGF-β1 that were attenuated by NAPc2 treatment starting 1 day after acute MI (Figure 12, B and C). In addition, NAPc2 improved cardiac function (Figure 12D), attenuated cardiac infiltration of CD11b^+^ myeloid cells (Supplemental Figure 18A), and diminished ERK1/2 phosphorylation, NOX2 expression, TGF-β1 activation, and the downstream target α-SMA in the infarcted heart (Figure 12E and Supplemental Figure 18B). Next, we investigated beneficial effects of NAPc2 in the adverse cardiac remodeling and the transition from non-reperfused MI to IHF. Compared with vehicle-treated mice, mice with short-term NAPc2 treatment from day 1 to day 7 post-MI showed significantly reduced cardiac fibrosis (Figure 12F) and improved cardiac function at day 28 after MI (Figure 12G). This protection from cardiac damage resulted in improved survival (Figure 12H). Collectively, our results show that targeting of TF signaling function on myeloid cells results in beneficial cardiac remodeling by limiting excess fibrosis, improves cardiac function, and attenuates the development of IHF after MI.

## Discussion

**Figure 13:**
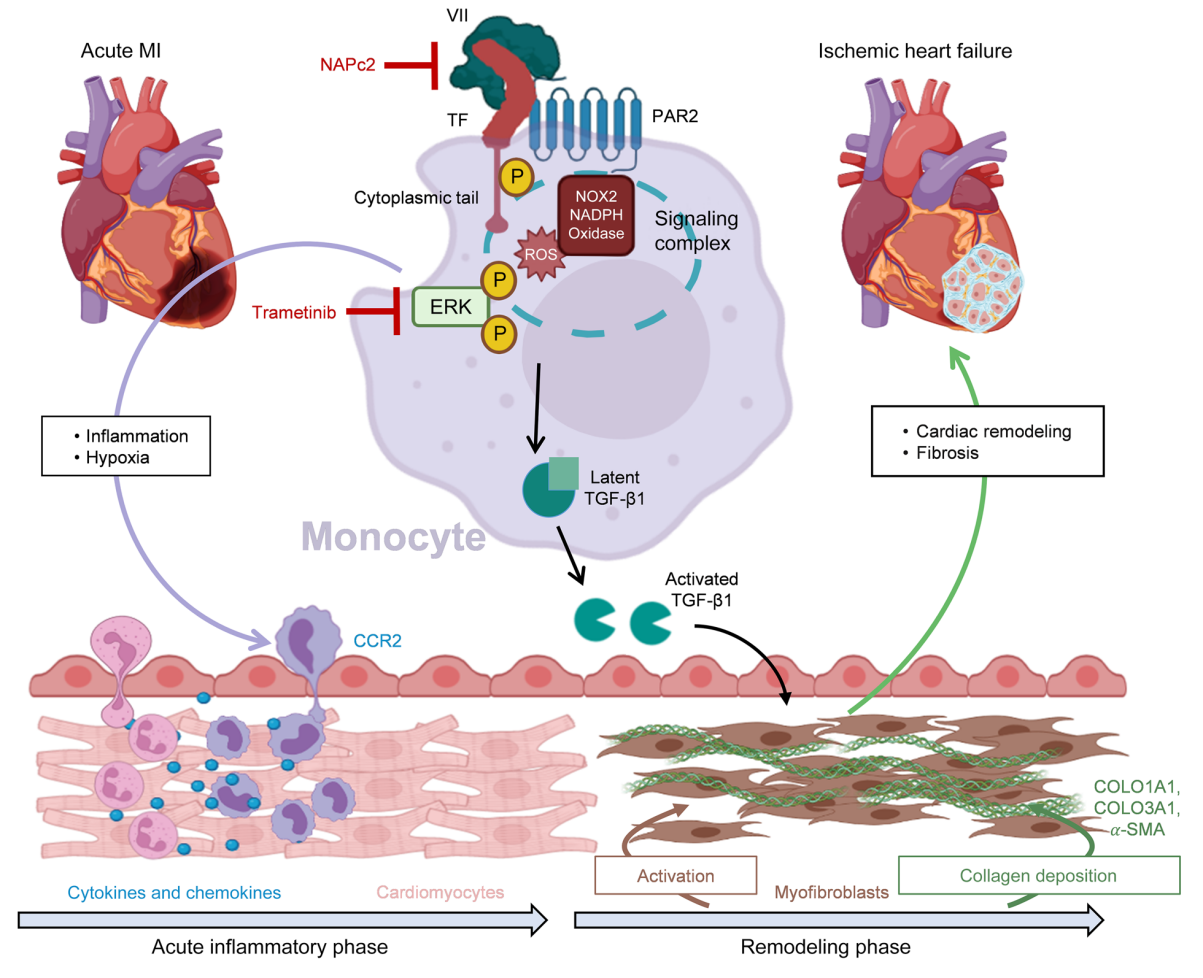
Schematic of proposed mechanism. CCR2, C-C chemokine receptor 2; COLO1A1, collagen type I α1 chain; COLO3A1, collagen type III α1 chain; ERK, extracellular signal–regulated kinase; NOX2, phagocyte NADPH oxidase; PAR2, protease-activated receptor 2; α-SMA, α-smooth muscle actin; TF, tissue factor.

With the presented experiments, we expand our view of the pathophysiology of coagulation and inflammation in MI and IHF and uncover a crucial function of coagulation-related signaling in adverse cardiac remodeling. Treatment of arterial thrombosis and vascular occlusion is central to the therapy of acute coronary syndromes. Here we provide novel insights into a non-canonical mechanism of coagulation in the infarcted myocardium beyond thrombosis, as well as TF-PAR2 signaling leading to TGF-β1 activation, and unravel the precise role of myeloid cells in this context (Figure 13).

Based on unbiased (phospho)proteomics in human IHF, MAPK1/ERK activation plays a crucial role in chronic ischemic heart disease. Inhibition of ERK1/2 activation in preclinical models of cardiac ischemia promotes angiogenesis (37) and reduces cardiomyocyte apoptosis (38, 39). We now show that activation of ERK1/2 during MI specifically occurs in myeloid cells infiltrating the ischemic heart and that intervention with the MEK inhibitor trametinib significantly diminishes the CCR2-dependent (40) Ly6C^hi^ monocyte recruitment into the infarcted myocardium. In addition, we establish a link between hyperactivation of the MAPK pathway and TGF-β1–mediated cardiac remodeling driven by inflammatory pro-oxidant myeloid cells infiltrating the infarcted heart.

Activation of TGF-β1 is required for its biological functions (41) and particularly for activating myofibroblasts in adverse cardiac remodeling after MI (42). We show with isolated cells that cytokine-primed monocytes exposed to hypoxia activate latent TGF-β1 dependent on NOX2 and MEK1/2 signaling. Furthermore, experiments with isolated monocytes from NOX2^–/–^ and PAR2^–/–^ mice in combination with in vivo analysis of myeloid cell–specific PAR2 and TF deletion confirmed that myeloid cell–derived TF-PAR2 signaling is the driver for TGF-β1 activation in MI. Disruption of the TF-PAR2 signaling complex on myeloid cells significantly reduces TGF-β1–mediated SMAD2 activation localized to myofibroblasts, which play a crucial role in post-MI remodeling (43, 44), but not to immune cells (28). These results demonstrate that the non-canonical signaling properties of coagulation factors in innate immune cells modulate profibrotic mesenchymal cells for tissue homeostasis in post-MI cardiac remodeling (45).

Platelet-derived TGF-β1 activation depends on reduced protein disulfide isomerase (46), which also plays a critical role in TF decryption (47–49) and signaling (24, 50). Notably, TF decryption is favored by complement activation (23, 24), which was a prominent feature of the cardiac tissue of IHF patients identified by our proteomic screen. Independent of clotting activation, the TF-FVIIa complex furthermore interacts with β_1_ integrin (51) and regulates ROS production by endosomal NOX2 trafficking, which depends on the TF cytoplasmic tail (26). ROS can regulate fibroblast proliferation and collagen synthesis in MI (52), but major cellular sources of ROS and underlying mechanisms of how they mediate cardiac remodeling in IHF have been elusive. Our presented data show that myeloid cell TF-PAR2 linked through the TF cytoplasmic tail is required for increased phagocyte-type NADPH oxidase–derived ROS production and TGF-β1 activation to promote cardiac remodeling and propagate IHF.

In the clinical setting, patients with coronary no-reflow and/or delayed presentation after onset of symptoms — so called subacute MI — show signs of thromboinflammation and are characterized by worse clinical outcome (33, 53, 54). However, biomarkers other than Q waves or T wave inversion in electrocardiography (55) are currently not established to predict poor outcomes of patients with MI that is not timely reperfused. Our data indicate that TF phosphorylation of circulating monocytes may serve as a marker for patients at increased risk of developing IHF and adverse remodeling following MI. In addition, our proof-of-principle experiments with pharmacological inhibitors delineate potential highly specific avenues to target this pathway. The putative clinical benefit is exemplified by NAPc2. This drug not only has dual antithrombotic action in the absence of excess bleeding (35) but, excitingly, disrupts signaling in TF-dependent coagulation, resulting in diminished fibrosis after experimental MI. The identified phosphorylation of the cytoplasmic tail of TF may serve as a biomarker applicable to liquid biopsies of patients with MI. It may facilitate clinical development of strategies to specifically target coagulation and TF-PAR2 signaling for myeloid cell reprogramming. This concept of interrupting coagulation-inflammatory signaling after MI has the potential to prevent TGF-β1 activation, to attenuate excess cardiac fibrotic damage, and to avert the development of IHF.

## Methods

### Human heart samples.

Ischemic heart samples were obtained from the left ventricular (LV) wall of explanted hearts after cardiac transplantation or from heart tissue obtained during implantation of an LV assist device. Donor hearts were used as controls and acquired from anonymized donors whose hearts could not be used for transplantation. Failing heart donors gave written informed consent for tissue donation. Storage and use of human heart tissue specimen by the Erich and Hanna Klessmann-Institute received approval of the ethics committee of the Ruhr-University Bochum, located in Bad Oeynhausen, Germany. Acquired heart samples were divided into at least 3 pieces, snap-frozen in liquid nitrogen, and stored at –80°C by HDZ-NRW.

### Proteomic profiling of human hearts.

Transmural heart tissue samples from 5 control hearts and 5 patients with IHF were lysed in 7 M urea, 2 M thiourea, 1% Phosphatase Inhibitor Cocktail 3 (Sigma-Aldrich), 100 mM NH_4_HCO_3_ by sonication at 4°C and centrifuged, and the protein concentration was determined with the Pierce protein assay (Thermo Fisher Scientific). For whole-proteome analysis, 20 μg was used for filter-aided sample preparation and 700 μg protein for in-solution digest followed by phosphopeptide enrichment. The proteins were processed following previously published protocol (56, 57). Purified peptides were reconstituted in 0.1% (vol/vol) formic acid for liquid chromatography–mass spectrometry (LC-MS) analysis on a nanoAcquity LC (Waters Corp.) (58). Eluting peptides were analyzed in positive-mode electrospray ionization MS by ion-mobility separation–enhanced data-independent acquisition (DIA) ultra-definition data-independent acquisition MS using elevated collision energy (UDMS^E^) on a Synapt G2-S HDMS mass spectrometer (Waters Corp.) as described previously (59). Acquired MS data were postacquisition lock mass corrected using [Glu1]-fibrinopeptide B. LC-MS DIA raw data processing was performed with ProteinLynx Global SERVER (PLGS) (version 3.02 build 5, Waters Corp.). The human reference proteomes (entries: 20,365; UniProtKB/Swiss-Prot) were used for peptide identification. The false discovery rate (FDR) for peptide and protein identification was assessed searching a reversed decoy database and set to a 1% threshold for database search in PLGS. Label-free quantification analysis was performed using ISOQuant as described previously (59). For each protein, absolute sample amounts were estimated using TOP3 quantification (60).

### Phosphopeptide analysis.

Protein was digested after reduction overnight with trypsin (TPCK-treated, Sigma-Aldrich), phosphopeptides were enriched on preloaded TiO_2_ spin tips, and bound phosphopeptides were eluted, desalted using Pierce graphite spin columns (Thermo Fisher Scientific), lyophilized, and reconstituted for LC-MS analysis on an Ultimate 3000 nanoUPLC (Thermo Fisher Scientific) and Nanospray Flex electrospray ionization source (Thermo Fisher Scientific). All samples were measured in triplicates. Mass-to-charge analysis of the eluting peptides was performed using an Orbitrap Exploris 480 (Thermo Fisher Scientific) in data-dependent acquisition mode. The MS proteomics data were deposited to ProteomeXchange via the PRIDE partner repository with the data set identifier PXD024727. Raw data processing for discovery phosphoproteomics analysis was performed in Proteome Discoverer v2.4 (Thermo Fisher Scientific) using Sequest HT Search Engine in the processing workflow. UniProtKB/Swiss-Prot entries of the human reference proteomes (entries: 20,365) were used as database for peptide and protein identification with maximum allowed missed cleavages of 2, and maximum precursor and fragment ion mass tolerance of 10 ppm and 0.02 Da, respectively. The phosphosite localization probabilities were determined using IMP-ptmRS (https://ms.imp.ac.at/index.php?action=ptmrs) with PhosphoRS mode enabled. Features were detected with Minora Feature Detector. In the consensus workflow, label-free quantification (LFQ) was performed by Feature Mapping and subsequent Precursor Ions Quantifier using the top 3 intense unique and razor peptides. Raw data processing for Integrative Inferred Kinase Activity (InKA) analysis was performed in MaxQuant v1.6.14.0 (61) using the same search engine settings as in Proteome Discoverer including LFQ with Match Between Runs enabled. The results were then submitted for InKA analysis (61).

The resulting quantitative information for proteins and phosphosites was analyzed using R including the packages tidyverse, pheatmap, and imputeLCMD. Fold changes (FCs) of IHF group versus control group were calculated using median intensity values. Two-sided *t* test assuming equal variances was performed for all samples that could be confidently identified with valid intensity values in at least 60%. All samples that were confidently appearing or disappearing in IHF condition (i.e., at least 60% identified intensities in one of the conditions and only missing values in the other condition) were assigned an FC of 100 and 0.01 as well as a *P* value of 0.001. The resulting *P* values were corrected for multiple testing using the Benjamini-Hochberg (FDR) method. The resulting proteins and phosphosites were then filtered for FCs greater than 2 and less than 0.5 and an adjusted *P* value less than 0.05. After sorting for descending FC, the protein and phosphopeptide abundances were log_10_-transformed, normalized by *z* score, and plotted as heatmap. Protein-protein interaction networks were generated using Cytoscape v3.7.2 including the STRING app, NetworkAnalyzer plug-ins, and ClueGO app (62–65). Changes in InKA scores were identified by calculation of FC and performance of *t* test applying the same settings as for proteome and phosphoproteome data. The log_2_ of the FC and –log_10_ of the adjusted *P* value were calculated and displayed in a volcano plot.

### Animals and in vivo treatments.

Nine- to 12-week-old male mice lacking 21 amino acids of the cytoplasmic tail of TF (TF^ΔCT^ mice) on a C57BL/6J background and PAR2^–/–^ mice on a C57BL/6N background were used along with strain-matched controls (26). PAR2^fl/fl^ mice were crossed to LysM^Cre^ mice to generate conditional knockout of PAR2 on the myeloid cell compartment (PAR2^fl/fl^ LysM^Cre+^ mice) (15, 66); as controls, Cre-negative PAR2^fl/fl^ littermates were used. Generation of TF^fl/fl^ LysM^Cre+/–^ mice has been previously described (67). All animals were bred and housed in the Translational Animal Research Center of the Johannes Gutenberg University, Mainz, Germany. For pharmacological interventions, C57BL/6J animals were used. LAD-ligated animals were further randomly divided into vehicle- or drug-treated groups. Trametenib (GSK1120212) was purchased from Selleck Chemicals and reconstituted in 200 μL vehicle (hydroxypropyl methylcellulose buffer) and orally administered to the mice (1 mg/kg/d) once a day starting from day 1 after MI throughout day 7. For NAPc2 treatment, mice were treated with i.p. injections (1 mg/kg/d) of NAPc2 reconstituted in NaCl physiological saline. Dosing was performed once a day starting from 1 day after MI until day 7.

### Mouse model of non-reperfused MI.

MI was induced by permanent ligation of the proximal LAD as described previously (68). Mice were anesthetized with medetomidine (500 μg/kg body weight), fentanyl (50 μg/kg body weight), and midazolam (5 mg/kg body weight). To antagonize the anesthesia, we injected atipamezole (2.5 mg/kg body weight) and flumazenil (0.5 μg/kg body weight). Sham surgery followed the same procedure except for ligation of the LAD. Mice received buprenorphine (0.075 mg/kg s.c.) twice daily for 2 days, starting on the day of surgery. Heart failure was defined in this study by a reduction of the LV ejection fraction (LVEF) below 35% and/or visual infarction of the LV. Animals that did not fulfill these criteria were excluded from the study.

### Bone marrow transplantation.

TF^ΔCT^, C57Ly5.1, and C57BL/6J mice aged 8–11 weeks were irradiated with a lethal dose of 9 Gy. Briefly, donor bone marrow (BM) was harvested in 2% PBS/FCS, filtered through a 70 μm cell strainer. Collected BM cells from the donor mice were washed in fresh 2% PBS/FCS and then resuspended at a final concentration of 4 × 10^8^ cells/mL. At 24 hours after irradiation, approximately 200 μL was injected into the recipient mice via the tail vein. Chimeric animals were allowed to recover for 9–10 weeks, followed by the LAD ligation. Donor versus host composition in the infarcted myocardium was determined by the flow cytometry analysis in the infarcted myocardium 7 days after MI.

### Echocardiography.

Transthoracic echocardiography was performed using a VEVO-3100 and VEVO-770 (FUJIFILM VisualSonics Inc.) High-Frequency Ultrasound System equipped with a 38 MHz (MX400) linear array transducer; images were acquired at a frame rate consistently above 200 frames. ECG and breathing rate were monitored, and body temperature was kept at 37°C using a heating system within the handling platform. Mice were examined longitudinally (from 1 day up to 4 weeks after MI) to measure LV end-diastolic volume (LVEDV), internal diameter in diastole and systole to calculate LVEDV and LVEF, posterior wall thickness in diastole, and interventricular septum thickness in diastole analyzed in parasternal long axis by means of M-mode linked to 2D B-mode images. Post-acquisition analysis was performed with Vevo LAB software (FUJIFILM VisualSonics Inc).

### Flow cytometry analysis of immune cells isolated from heart and blood.

Infiltration of immune cells into the infarcted myocardium was analyzed by flow cytometry. Infarcted myocardium was enzymatically digested with collagenase II (1 mg/mL)/DNase I (50 μg/mL) for 30 minutes at 37°C. The lysates were passed through a 70 μm cell strainer and washed with 2% PBS/FCS. Cells were blocked with FC blocking solution (eBioscience/Invitrogen) (anti-CD16/anti-CD32) and stained with the following monoclonal antibodies or reagent: CD45 APC–eFluor 780 (eBioscience, clone 30-F11); CD45.1 APC–eFluor 780 (eBioscience, clone A20); CD45.2 FITC (eBioscience, clone 104); B220 FITC (eBioscience, clone RA3-6B2); CD11b FITC (BD Biosciences, clone M1/70) or PerCP-Cy5.5 (eBioscience, clone M1/70); CD90.2 SuperBright 645 (eBioscience, clone 53-2.1); NK1.1 PE-Cy7 (eBioscience, clone PK136); Ly6G PE (BD Biosciences, clone 1A8); Ly6C Pacific blue (eBioscience, clone HK1.4); F4/80 APC (eBioscience, clone BM8); TF PE (R&D Systems); Viability Dye eFluor 506 (eBioscience). Cells were analyzed on the Attune NxT Flow Cytometer (Thermo Fisher Scientific). Living cells, including CD45^+^CD90.2^–^CD220^–^NK1.1^–^CD11b^+^Ly6G^+^ neutrophils, CD45^+^CD90.2^–^CD220^–^NK1.1^–^CD11b^+^Ly6G^–^F4/80^–^Ly6C^hi^ inflammatory monocytes, CD45^+^CD90.2^–^CD220^–^NK1.1^–^CD11b^+^Ly6G^–^F4/80^–^Ly6C^lo^ reparative monocytes, and CD45^+^CD90.2^–^CD220^–^NK1.1^–^CD11b^+^F4/80^+^ macrophages were analyzed by FlowJo software (FlowJo version 10, BD Biosciences).

### PBMC and monocyte isolation and in vitro culture.

Venous blood was drawn by right atrial cardiac puncture from mice, and PBMCs were isolated by Histopaque (catalog 11191, 1077, Sigma-Aldrich) gradient cell separation. Peripheral blood collected from the patients was added 1:1 to Histopaque-1119 and Histopaque-1077 (catalog 11191, 1077, Sigma-Aldrich). After centrifugation at 700*g* for 30 minutes at room temperature, the mononuclear cell layer was washed with PBS for platelet elimination and analyzed for living CD45^+^TF^+^ leukocytes and CD45^+^TF^+^CD11b^+^CD115^+^Ly6C^+^ and CD45^+^TF^+^CD11b^+^CD115^+^Ly6C^–^ monocytes. Monocytes were isolated from PBMCs by negative selection using Monocyte Isolation Kit II (human: catalog 130-117-337, Miltenyi Biotec) according to the manufacturer’s instructions and blocked with 1% BSA followed by primary antibody incubation with phospho-specific mouse anti–human TF antibody (4G6) (32) and mouse anti–human TF antibody (10H10). After overnight incubation, sections were counterstained with the secondary antibodies donkey anti–rabbit IgG (ab150076, Abcam), goat anti–rat IgG (ab150160, Abcam), and goat anti–mouse IgG (ab150116, Abcam) for 1 hour and mounted in antifading mounting medium (P36962, Thermo Fisher Scientific) for confocal laser scanning. BM cell suspensions were isolated by flushing of femurs and tibiae of 8- to 12-week-old mice and passed through a 70 μm nylon strainer to remove the cell debris. Monocytes were isolated with the Mouse Monocyte Isolation Kit (STEMCELL Technologies Inc.). To mimic in vivo ischemic conditions, a protocol of oxygen-glucose deprivation (OGD) was applied along with the cytokine cocktail mix (IL-6, TNF-α, and MCP-1) at concentrations of 20 ng/mL for 4 hours followed by Western blotting for p-ERK1/2, NOX2, and TGF-β1. For experiments with ERK1/2 inhibition, primary mouse monocytes were pretreated for 1 hour with trametinib (10 μM) followed by the OGD along with the cytokine cocktail mix. For blocking of TF-FVIIa signaling on human monocytes, isolated monocytes from healthy individuals were treated with NAPc2 in the presence of OGD along with the cytokine cocktail mix followed by the protein expression analysis of p-ERK1/2, NOX2, and TGF-β1.

### Dihydroethidium-HPLC.

Minced myocardial tissue was incubated with 50 μmol/L of dihydroethidium (DHE) at 37°C for 30 minutes, washed, and extracted for the superoxide-specific (O_2_^•−^-specific) oxidation product 2-hydroxyethidium by HPLC. The system consisted of a control unit, 2 pumps, a mixer, detectors, a column oven, a degasser, an autosampler (AS-2057 Plus, Jasco), and a C18-Nucleosil 100-3 (125 × 4) column (Macherey-Nagel). DHE was detected by its absorption at 355 nm, whereas 2-hydroxyethidium and ethidium were detected by fluorescence (excitation 480 nm/emission 580 nm).

### Confocal microscopy.

Cryosections of the myocardium (5–8 μm) were fixed with 4% paraformaldehyde and permeabilized with 0.1%–0.2% Triton X-100 for 10 minutes, blocked with 5% BSA, and costained with the rabbit polyclonal antibody raised against a synthetic peptide containing phosphorylated Ser and Thr residues adjacent to the conserved Pro residue of cytoplasmic TF (32), and anti-CD45 (ab10558, Abcam). To determine p-ERK1/2^+^ cells, anti–p-ERK1/2 (4370S, Cell Signaling Technology) with costaining for anti-CD31 (sc-18916, Santa Cruz Biotechnology), anti-CD45 (30-F11, BioLegend), anti–α-SMA (A2547, Sigma-Aldrich), and anti–cardiac troponin T (ab92546, Abcam) was used. Anti-NOX2 (611414, BD Biosciences), anti-CD68 (ab955, Abcam), and anti–TGF-β1 (NBP2-22114, Novus Biologicals) were used to monitor NOX2 and TGF-β1 localization. We assessed cardiomyocyte apoptosis using a fluorescence-labeled TUNEL kit (12156792910, Roche) counterstained for anti–cardiac troponin T (ab92546, Abcam). To analyze the abundance of p-SMAD2^+^ cells in the infarcted myocardium, p-SMAD2 (44-244g, Invitrogen) with costaining for α-SMA (A2547, Merck) and anti-CD45 (30-F11, BioLegend) was used. The confocal microscopy was performed on a Leica SP8 Confocal Microscope with a ×20 0.75 NA dry objective with sequential scanning and detection, with 405 nm excitation and 411–488 nm detection for DAPI, 488 nm excitation and 508–570 nm detection of Alexa Fluor 488, 552 nm excitation and 590–650 nm detection of Alexa Fluor 594, and 638 nm excitation and 646–720 nm detection of Alexa Fluor 647. Images of 512 × 512 pixels (up to 5,280 × 5,280 pixels) in 580 × 580 μm regions were acquired in mosaic fashion with 10% of overlap between neighboring images to allow for image stitching and with axial stacks of the entire tissue height with 2 μm steps for each imaging area with Leica Navigator software. The mosaic images were then merged with a smoothing operation of the 10% overlapping regions to create the fluorescence images of the tissue cryosections. At least 3 individual images were acquired of each section. Quantification using Fiji/ImageJ (NIH) (with the specific Coloc2 and Tunel plug-ins) was corroborated with Leica LASX or Imaris software (version 9.3.1, Bitplane).

### Western blotting.

PBMCs, monocytes, or cardiac tissue were homogenized in lysis buffer (1% Triton X-100, 20 mM Tris pH 7.4–7.6, 150 mM NaCl, 50 mM NaF, 1 mM EDTA, 1 mM EGTA, 1 mM glycerol phosphate, 1% SDS, 100 mM PMSF, and 0.1% protease/phosphatase inhibitor cocktail) for 20 minutes on ice. Lysates were cleared by centrifuging at 11,000*g* for 15 minutes at 4°C. Total protein concentration was estimated using Lowry Assay (DC Protein Assay, Bio-Rad), and an equal protein amount in all samples was mixed in 6× Laemmli sample buffer, heated to 99°C for 10 minutes, separated according to their molecular weight in an SDS-PAGE gel (4%–15%), and probed with respective primary antibodies against p-ERK1/2 (4370S, Cell Signaling Technology), ERK1/2 (4695, Cell Signaling Technology), p-p38 (4511S, Cell Signaling Technology), p38 (9219, Cell Signaling Technology), TGF-β1 (NBP2-22114, Novus Biologicals), p-SMAD2, SMAD2 (12747T, Cell Signaling Technology), p67^phox^ (610912, BD Biosciences), NOX2 (611414, BD Biosciences), and α-SMA (ab7817, Abcam). For human monocytes and myocardial biopsies, phospho-specific mouse anti–human TF antibody (4G6) and mouse anti–human TF antibodies (10H10) were used. After overnight incubation with the primary antibodies, PVDF membranes were incubated with secondary antibodies for 2 hours (goat anti–rabbit HRP, 7074, Cell Signaling Technology; and anti–mouse HRP, 7076, Cell Signaling Technology) and developed using Fusion FX (PEQLAB Biotechnologie GmbH) and Western blotting ECL (Thermo Fisher Scientific) chemiluminescent reagents. Relative densitometry was performed with appropriate software, and the ratios were used for statistical analysis.

### Quantitative reverse transcription PCR.

RNA from pulverized heart samples was extracted by guanidine isothiocyanate phenol chloroform extraction. Relative mRNA expression analysis of chemokines and cytokines was performed by quantitative real-time reverse transcription PCR (qRT-PCR). qRT-PCR examination was performed with the QuantiTect Probe RT-PCR kit (Qiagen) using 0.05 μg of total RNA. For cDNA synthesis, 1 μg of RNA was used. The qPCR buffer in each well was composed of 10 μL 2× Master Mix (Applied Biosystems), 5 μL RNase-, DNase-, and protease-free purified water, 1 μL primer of the gene being investigated, and 5 μL of the cDNA sample. The following primers were used: *COLO1A1* (forward GCTCCTCTTAGGGGCCACT, reverse CCACGTCTCACCATTGGGG), *COLO3A1* (forward CTGTAACATGGAAACTGGGGAAA, reverse CCATAGCTGAACTGAAAACCACC), *ACTA2* (forward GTCCCAGACATCAGGGAGTAA, reverse TCGGATACTTCAGCGTCAGGA), and *Posn* (forward CCTGCCCTTATATGCTCTGCT, reverse AAACATGGTCAATAGGCATCACT). TaqMan Gene Expression assays (Applied Biosystems) for TATA-box binding protein (*Tbp*; Mm00446973_m1), *Ccr2* (Mm00438270_m1), *Il6* (Mm00446190_m1), *Ccl2* (Mm00441242_m1), and *Tnf* (Mm00443260_g1) were used. The relative mRNA expression level quantification was carried out according to the ΔΔCt method and normalized to the reference gene (*Tbp*).

The following primers were used for human heart samples: *IL6* (forward ACAAGCGCCTTCGGTCCAG, reverse CAATCTGAGGTGCCCATGCTA), *CCR2* (forward TGCCTGAGACAAGCCACAAG, reverse GGTGACCGTCCTGGCTTTTA), and *CCL2* (forward AAGATCTCAGTGCAGAGGCTCG, reverse TTGCTTGTCCAGGTGGTCCAT); and *GAPDH* (forward GACAGTCAGCCGCATCTTCT, reverse GCGCCCAATACGACCAAATC) was used for normalization of the data.

### Clinical study.

Twelve patients enrolled in the MICAT study (Mainz Intracoronary Database, ClinicalTrials.gov NCT02180178) were examined. Subacute MI was defined as follows: elevated circulating cardiac troponin I compatible with myocardial injury according to the Fourth Universal Definition of MI (69); symptoms of acute coronary syndrome >24 hours to <30 days prior to percutaneous coronary intervention; signs of subacute MI in electrocardiogram. Written informed consent was obtained from every participant. Medical history was documented, and body weight, height, and heart rate were obtained. Thirty milliliters of venous blood drawn from the cubital vein of the right arm was used for monocyte isolation.

### Statistics.

Statistical analysis was performed with Prism software, version 8 (GraphPad Software Inc.). The results are presented as mean ± SEM. First, the Shapiro-Wilk and Kolmogorov-Smirnov normality tests were used to determine whether the data were normalized. In the case of a normal distribution, a 2-sided *t* test was used for the comparison of 2 experimental groups, and an ordinary 1-way ANOVA followed by a Šidák’s multiple-comparison test was performed for more than 2 experimental groups. The 2-way ANOVA with Bonferroni’s post hoc test was used for more than 2 test groups and more than 1 measurement time. In the absence of a normal distribution, 2 test groups were evaluated by a Mann-Whitney test. For more than 2 experimental groups, a Kruskal-Wallis test was performed, followed by Dunn’s test for multiple comparisons. Asterisks are used as follows: *P < 0.05; **P < 0.01; ***P < 0.001. Pearson’s correlation coefficient was used to indicate the ratio between the covariance of 2 variables and the product of their standard deviations, with a value +1 indicating perfect positive correlation and a value of 0 implying that there is no linear dependency between the variables.

### Study approval.

All animal experiments were carried out in accordance with the *Guide for the Care and Use of Laboratory Animals* (National Academies Press, 2011) and approved by the Landesuntersuchungsamt Rheinland-Pfalz (Koblenz, Germany). The protocol of the MICAT study (Mainz Intracoronary Database, ClinicalTrials.gov NCT02180178) was approved by the local ethics committee of the state of Rhineland-Palatinate, Germany. Written informed consent was obtained from each participant.

## Author contributions

VG designed and performed experiments, analyzed data, and prepared the manuscript draft. MM, T Michna, SF, RJ, JL, PE, JW, MK, SW, KVM, and ST designed and analyzed experiments. GSH assisted in designing, conducting, and analyzing imaging experiments. T Münzel, AD, MB, and SK provided assistance in writing. HM and TG obtained patient samples and collected clinical data. WR provided crucial materials and guidance for their use in the study, conceptualized experiments, and made critical revisions to the manuscript. PW conceptualized, coordinated, and supervised the study, interpreted experiments, and wrote the manuscript.

## Supplementary Material

Supplemental data

Supplemental table 1

Supplemental table 2

Supplemental table 3

## Version 1 12/22/2022

In-Press Preview

## Version 2 02/15/2023

Electronic publication
