# Supplementary material for: Targeting myeloid cell coagulation signaling blocks MAP kinase/TGF-β1–driven fibrotic remodeling in ischemic heart failure
Source: J Clin Invest. 2023 Feb 15;133(4):e156436. doi: 10.1172/JCI156436 (PMC9927945; doi:10.1172/JCI156436)
Supplement: Supplemental data [file jci-133-156436-s159.pdf]

Supplementary Materials

Targeting myeloid cell coagulation signaling blocks MAP kinase/TGF-β1 drives fibrotic remodeling in ischemic heart failure.

Venkata Garlapati [1,2,3], Michael Molitor [1,2,3], Thomas Michna [4], Gregory Harms [4], Stefanie Finger[1,2], Rebecca Jung [1,2,5], Jeremy Lagrange [1], Panagiotis Efentakis [1], Johannes Wild [1,2,3], Maike Knorr[1,2], Susanne Karbach MD[1,2,3] , Sabine Wild [1,2,3], Ksenija Vujacic-Mirski [2], Thomas Münzel [1,2,3] , Andreas Daiber [2,3], Moritz Brandt [1,2,3], Tommaso Gori [2,3], Hendrik Milting [6] , Stefan Tenzer [4], Wolfram Ruf [1,3,7], Philip Wenzel [1,2,3,8]

[1] Center for Thrombosis and Hemostasis, [2] Department of Cardiology, University Medical Center Mainz, [3] German Center for Cardiovascular Research (DZHK) – Partner site Rhine-Main, [4] Institute of immunology, [5] Institute for Molecular Medicine, University Medical Center Mainz, Langenbeckstr. 1, 55131 Mainz, [6] Erich und Hanna Klessmann-Institut für Kardiovaskuläre Forschung und Entwicklung, Herz- und Diabeteszentrum NRW, Georgstr. 11 D-32545 Bad Oeynhausen, [7] Department of Immunology and Microbiology, Scripps Research, 10550 North Torrey Pines Road, La Jolla, CA 92037, USA [8] Department of Biochemistry, Cardiovascular Research Institute Maastricht School for Cardiovascular Diseases (CARIM), Maastricht University, Maastricht, the Netherlands

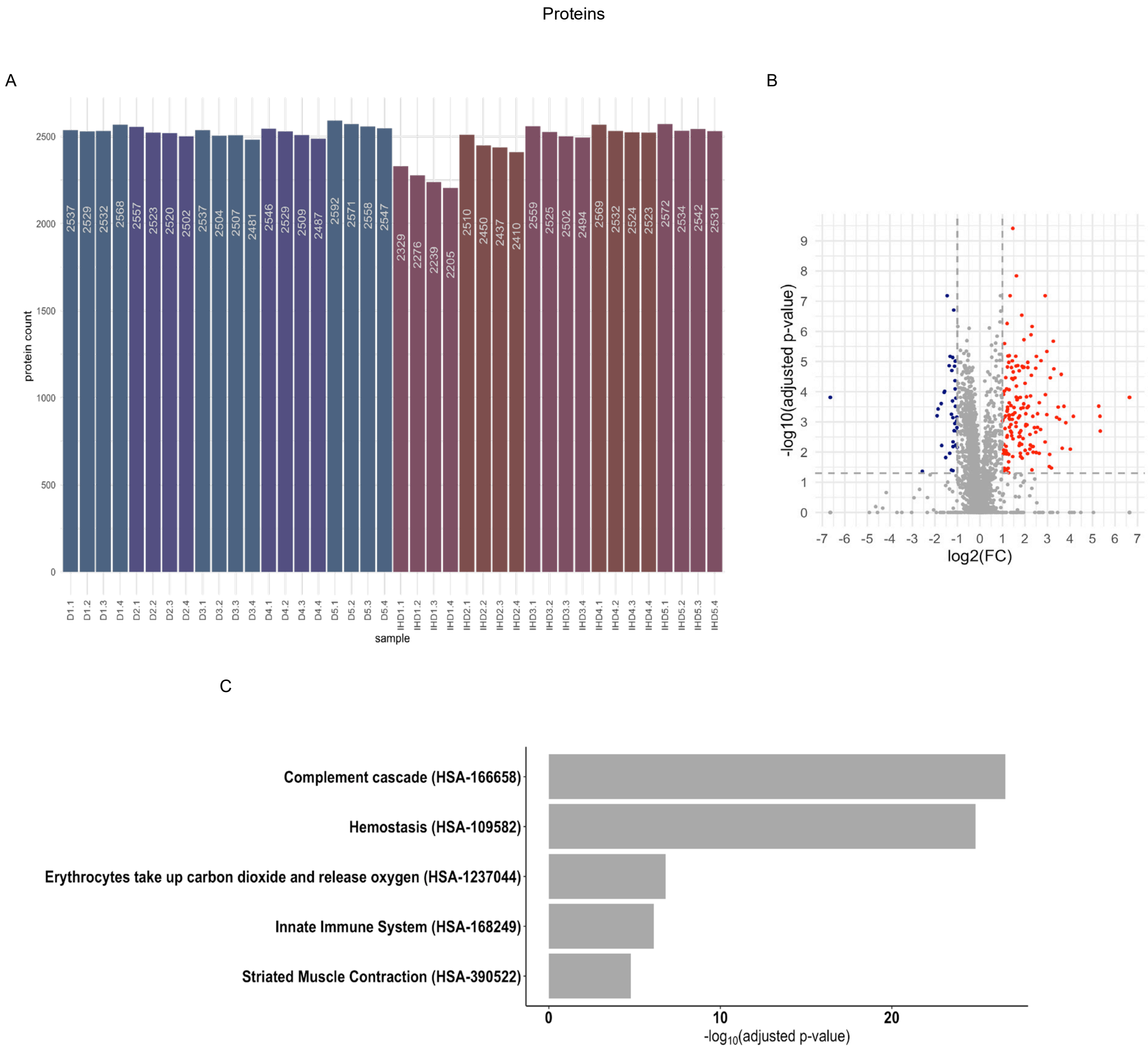

Supplementary Figure 1. A) Number of identified proteins across all biological and technical replicates. **B)** Volcano plot showing the fold changes and -$\log_{10}$ (adjusted p-values) on proteome level. **C)** Reactome enrichment terms for upregulated proteins without significant enrichment of downregulated proteins.

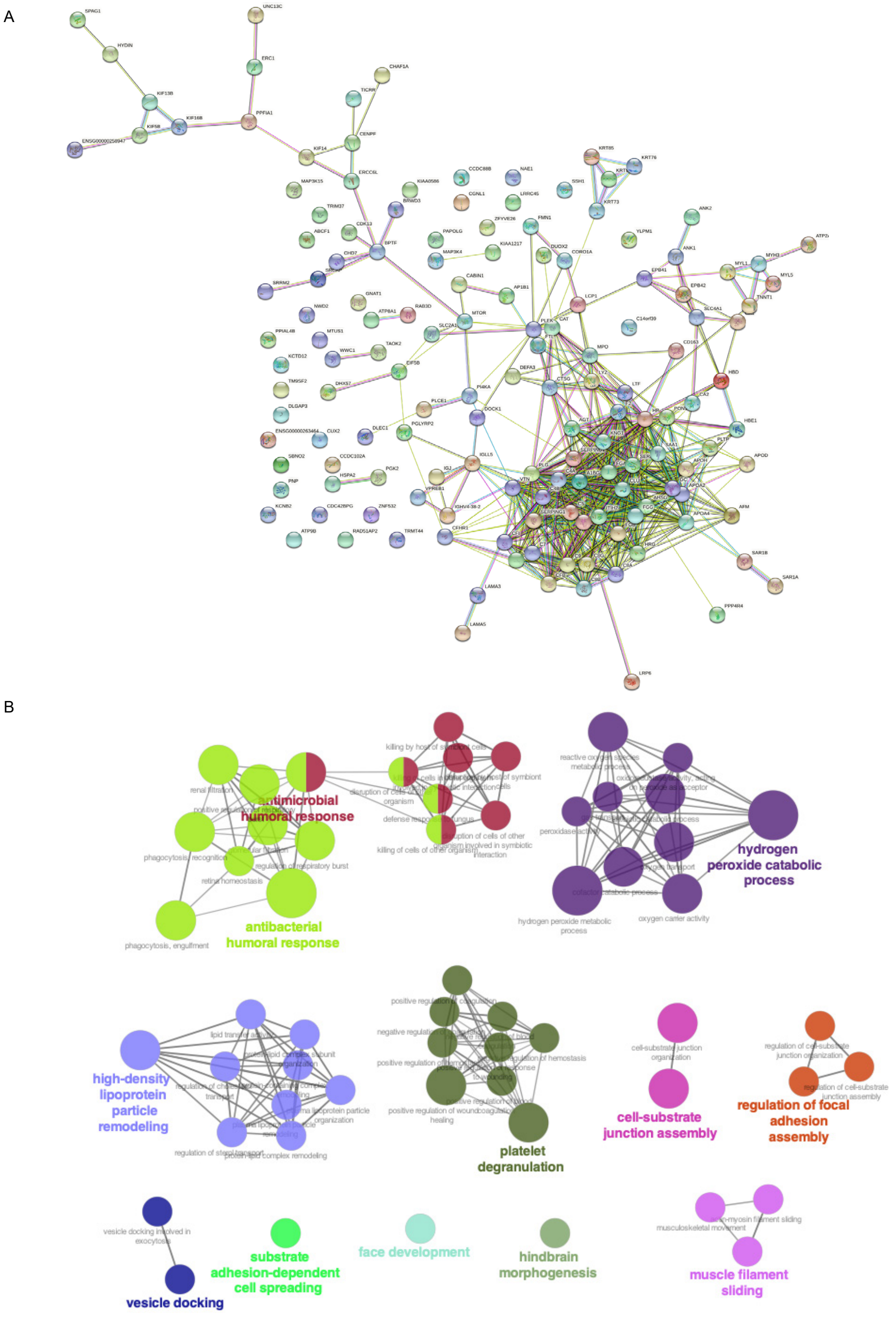

Supplementary Figure 2. A) Protein-protein interaction network for all upregulated proteins obtained from STRING-DB. **B)** Gene ontology term clustering for all upregulated proteins obtained from Cytoscape ClueGo app.

A

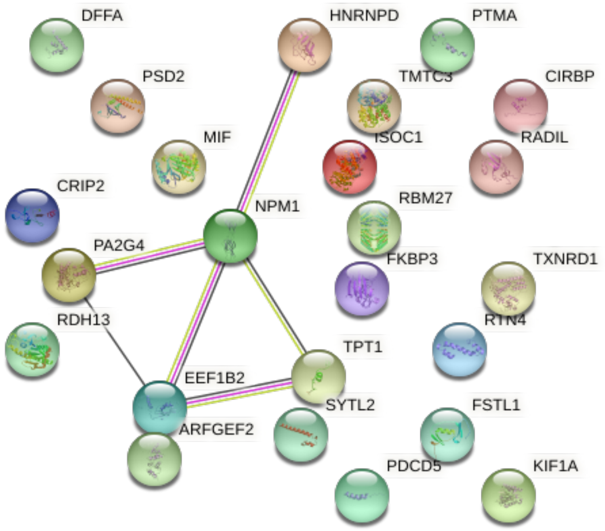

B

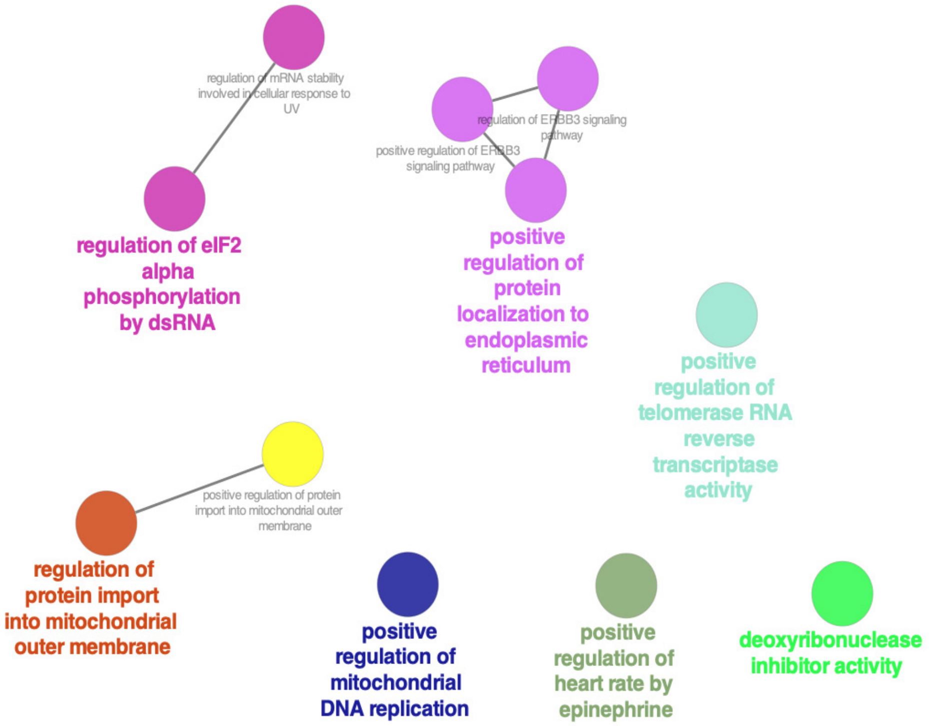

Supplementary Figure 3. A) Protein-protein interaction network for all downregulated proteins obtained from STRING-DB. **B)** Gene ontology term clustering for all downregulated proteins obtained from Cytoscape ClueGo app.

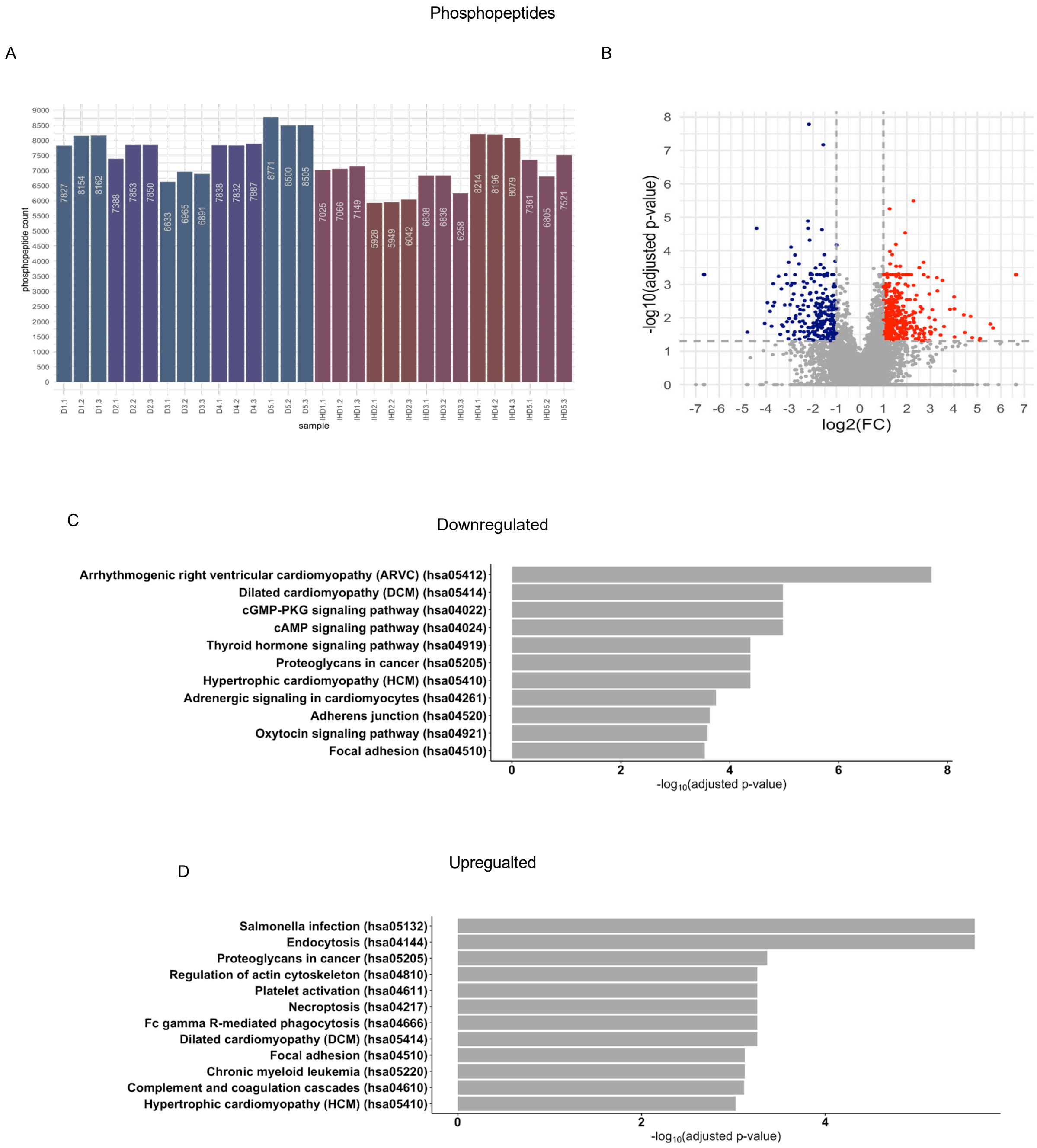

Supplementary Figure 4. A) Number of identified phosphopeptides across all biological and technical replicates. **B)** Volcano plot showing the fold changes and -$\log_{10}$ (adjusted p-values) on phosphopeptide level. **C)** Reactome enrichment terms for downregulated and **D)** phosphopeptides.

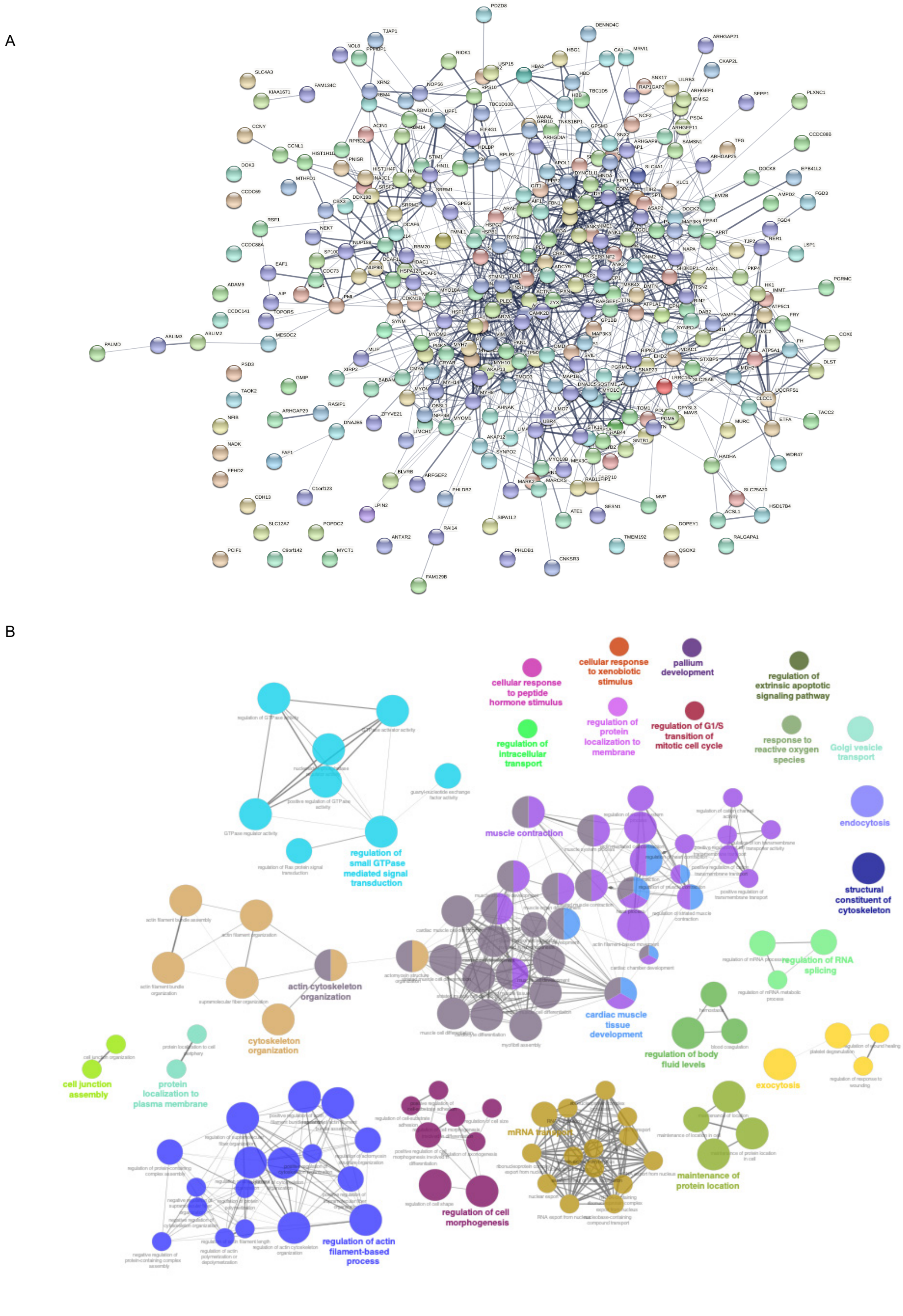

Supplementary Figure 5. A) Protein-protein interaction network for all upregulated phosphoproteins obtained from STRING-DB. **B)** Gene ontology term clustering for all upregulated phosphoproteins obtained from Cytoscape ClueGo app.

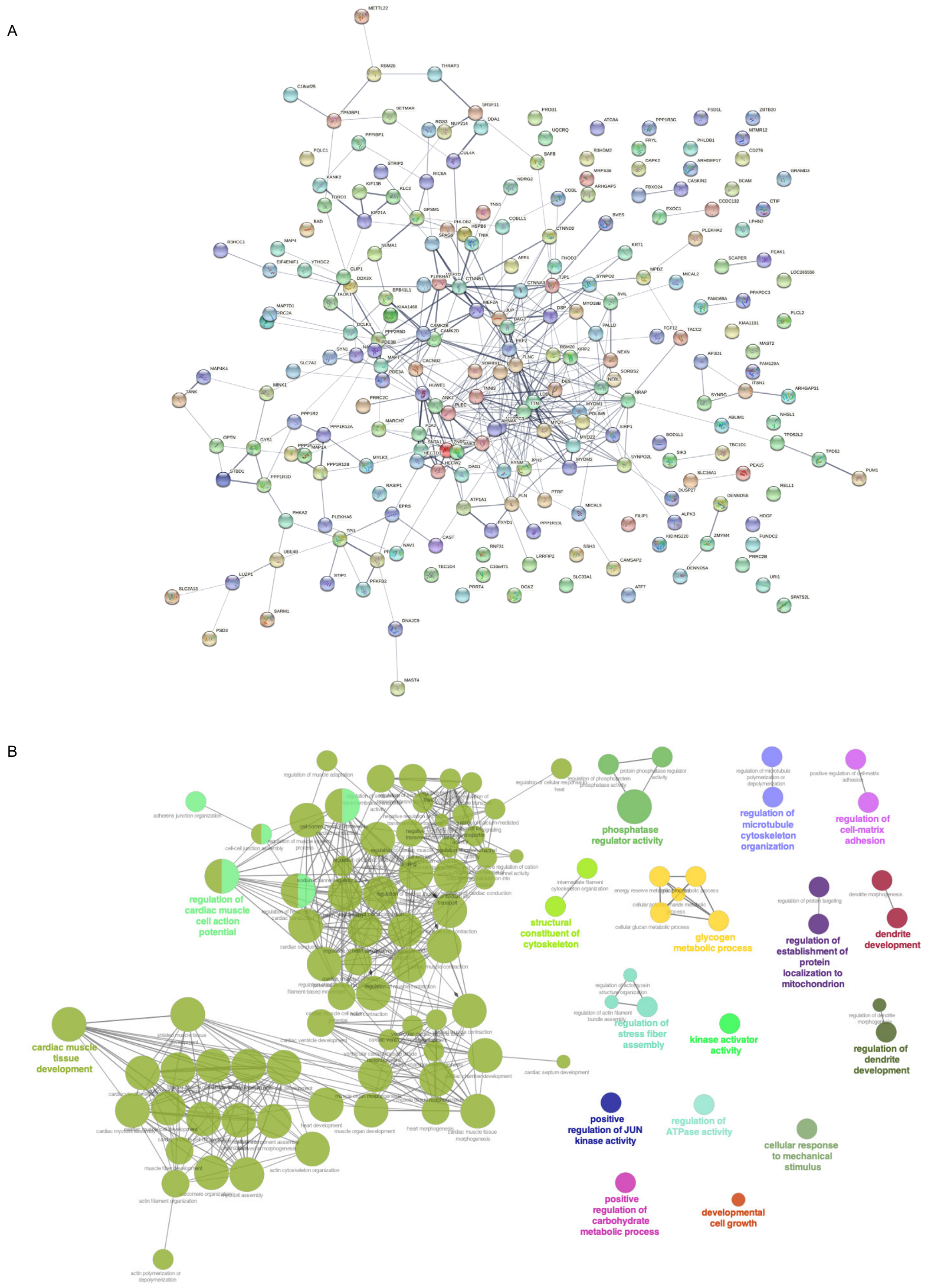

Supplementary Figure 6. A) Protein-protein interaction network for all downregulated phosphoproteins obtained from STRING-DB. **B)** Gene ontology term clustering for all downregulated phosphoproteins obtained from Cytoscape ClueGo app.

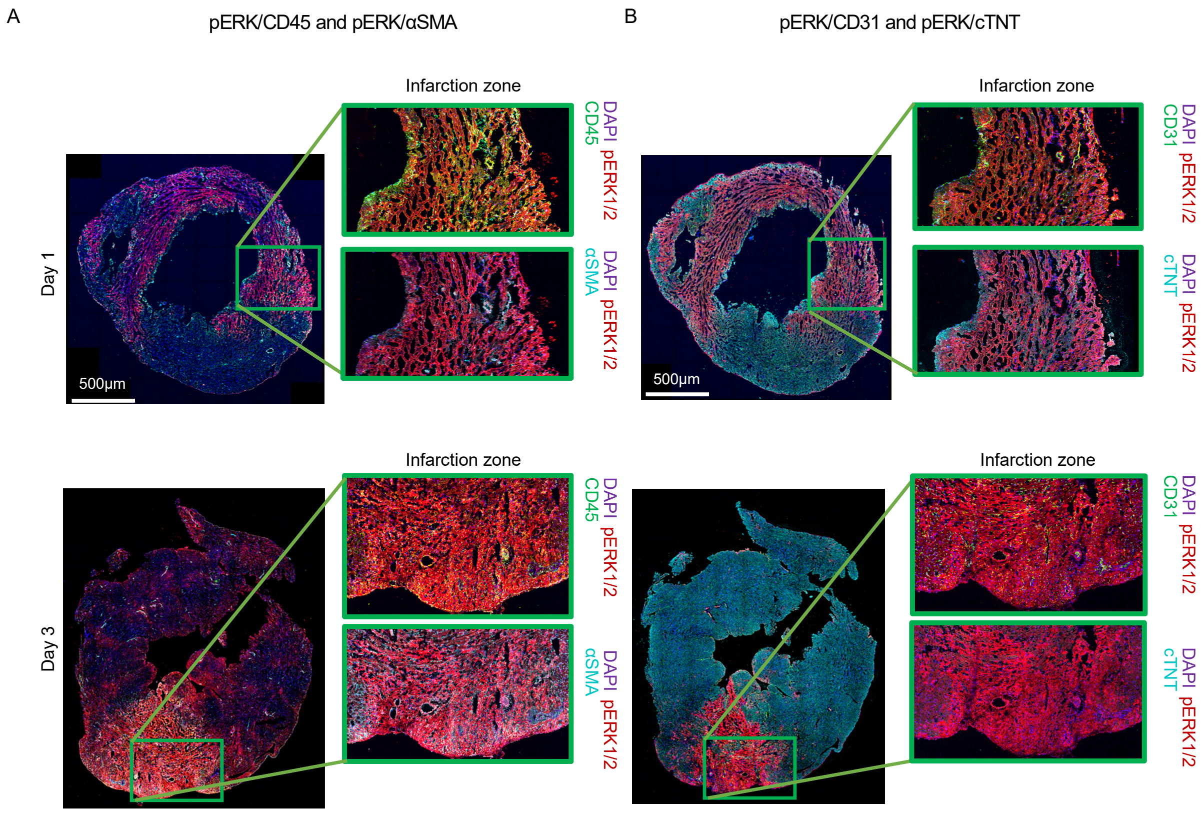

Supplementary Figure 7. Representative confocal images of myocardial cryo-sections obtained from WT (C57BL/6J) mice at day 1 and day 3 after MI stained for pERK1/2 and **A)** CD45 or αSMA; **B)** CD31 or cTNT.

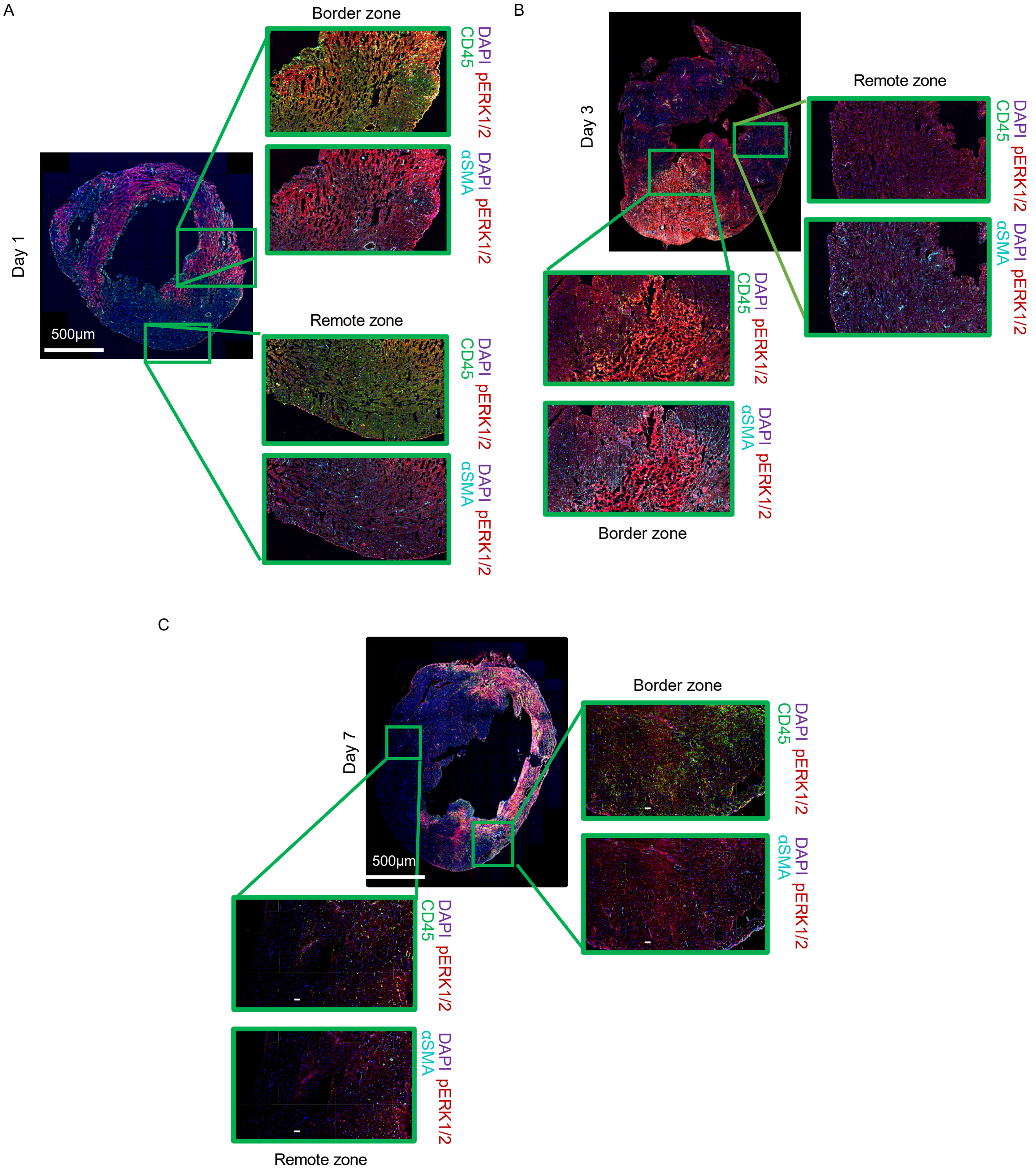

Supplementary Figure 8. Representative confocal images for the border and remote zones stained for pERK1/2 and co-stained for CD45 or αSMA on **A)** day 1, **B)** day 3, and **(C)** day 7 after MI. Quantitative image analysis for these experiments is presented in Figure 2A.

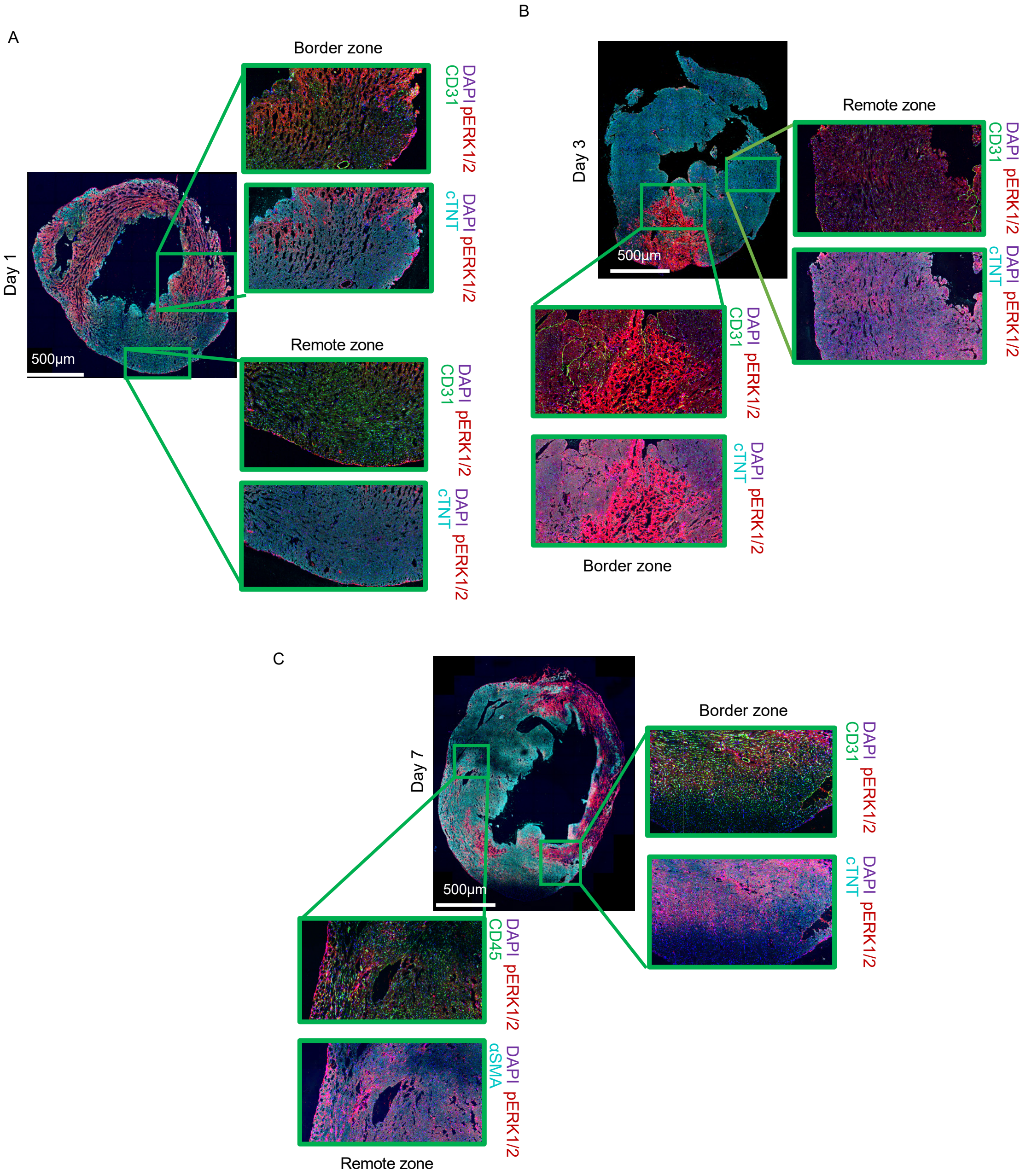

Supplementary Figure 9. Representative confocal images for the border and remote zones stained for pERK1/2 and co-stained for CD31 or cTNT on **A)** day 1, **B)** day 3, and **(C)** day 7 after MI. Quantitative image analysis for these experiments is presented in Figure 2A.

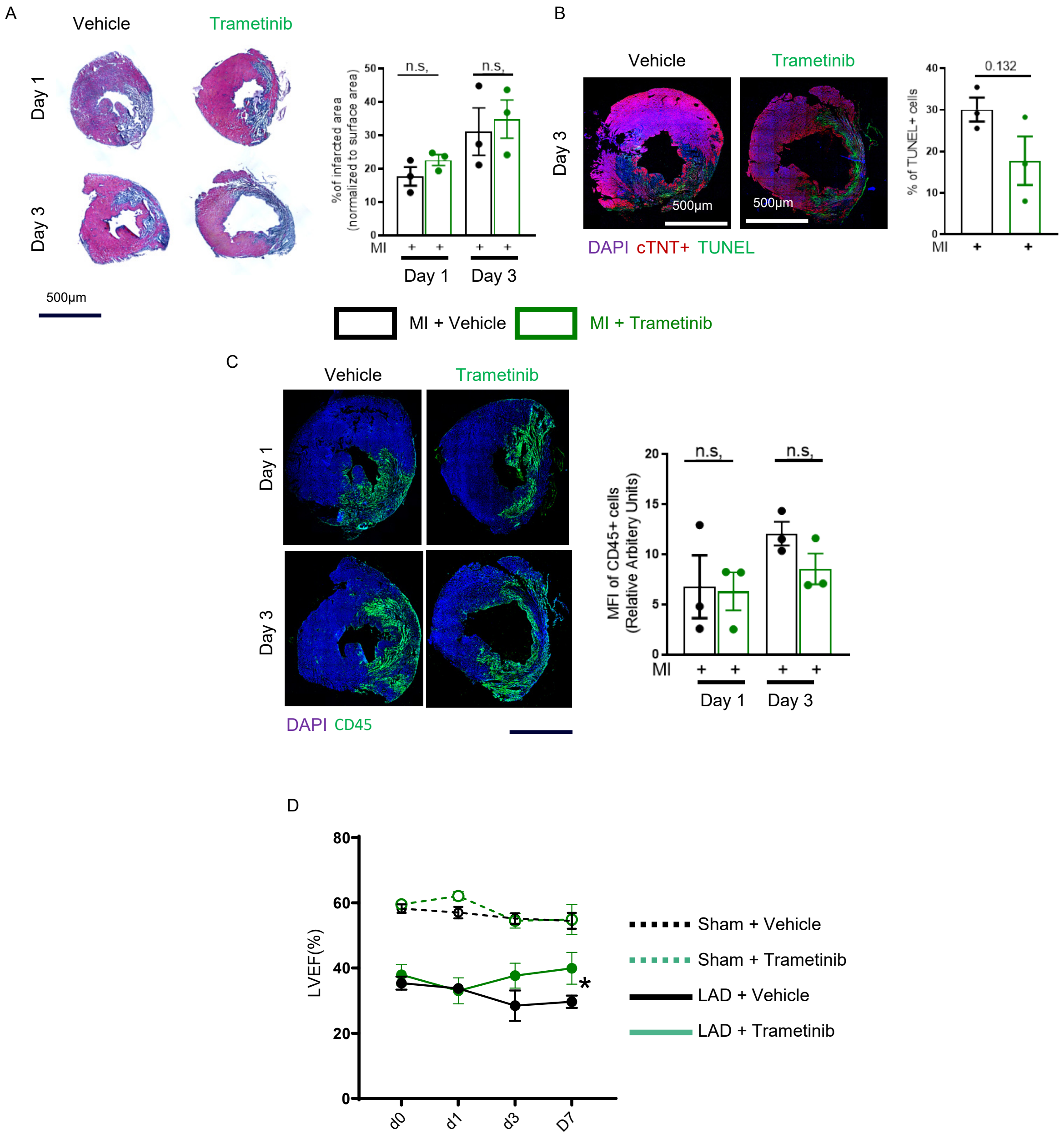

Supplementary Figure 10. A) Representative images and quantification of infarct size with Masson's Trichrome staining obtained from vehicle or trametinib treated animals (1 mg/kg/d) at day 1 and 3; n = 3; Mann-Whitney test, **B)** Representative confocal images and quantification of TUNEL-positive cardiomyocytes in the infarcted myocardium counterstained for DAPI after 3 days post MI; n = 3; Mann-Whitney t test. **C)** Representative confocal images and quantification of CD45^{+} cells in the infarcted myocardium obtained from vehicle or trametinib treated animals (1 mg/kg/d) at day 1 and 3; n = 3; Mann-Whitney test**. D)** Longitudinal echocardiographic studies of LVEF (%) in PLAX M-mode; n = 5-7; two-way ANOVA, Bonferroni's multiple comparisons test. Data are shown as mean ± SEM.

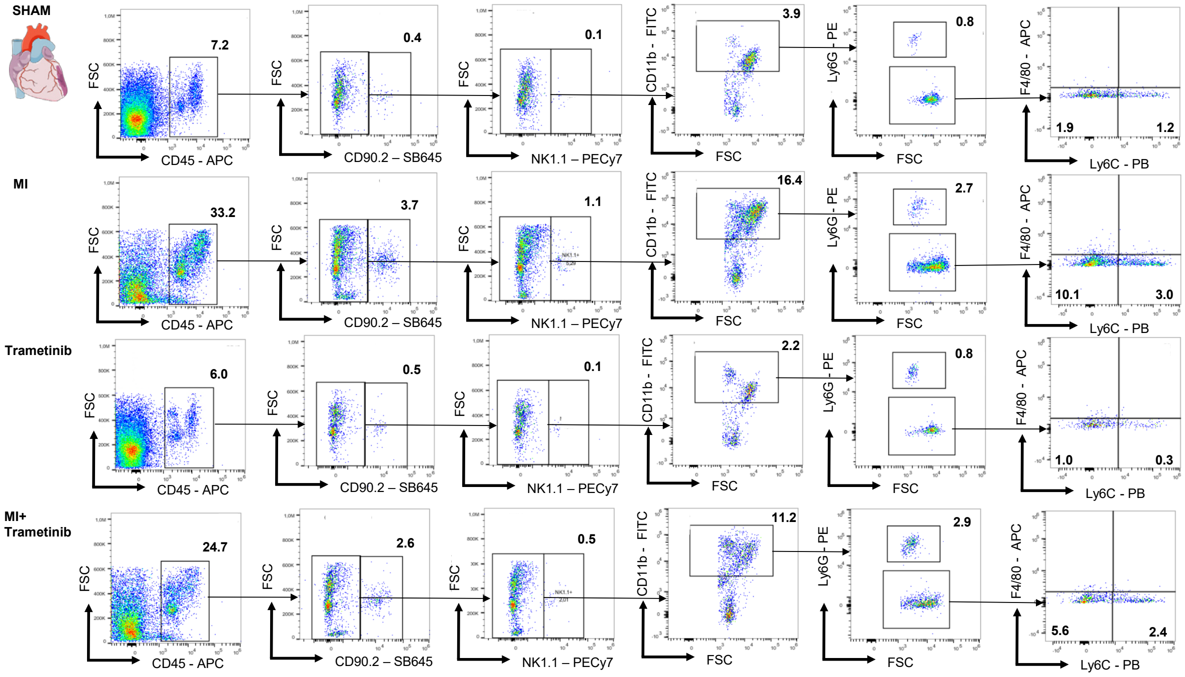

Supplementary Figure 11. Representative FACS gating strategy for the identification of major immune cells shown in Figure 3B.

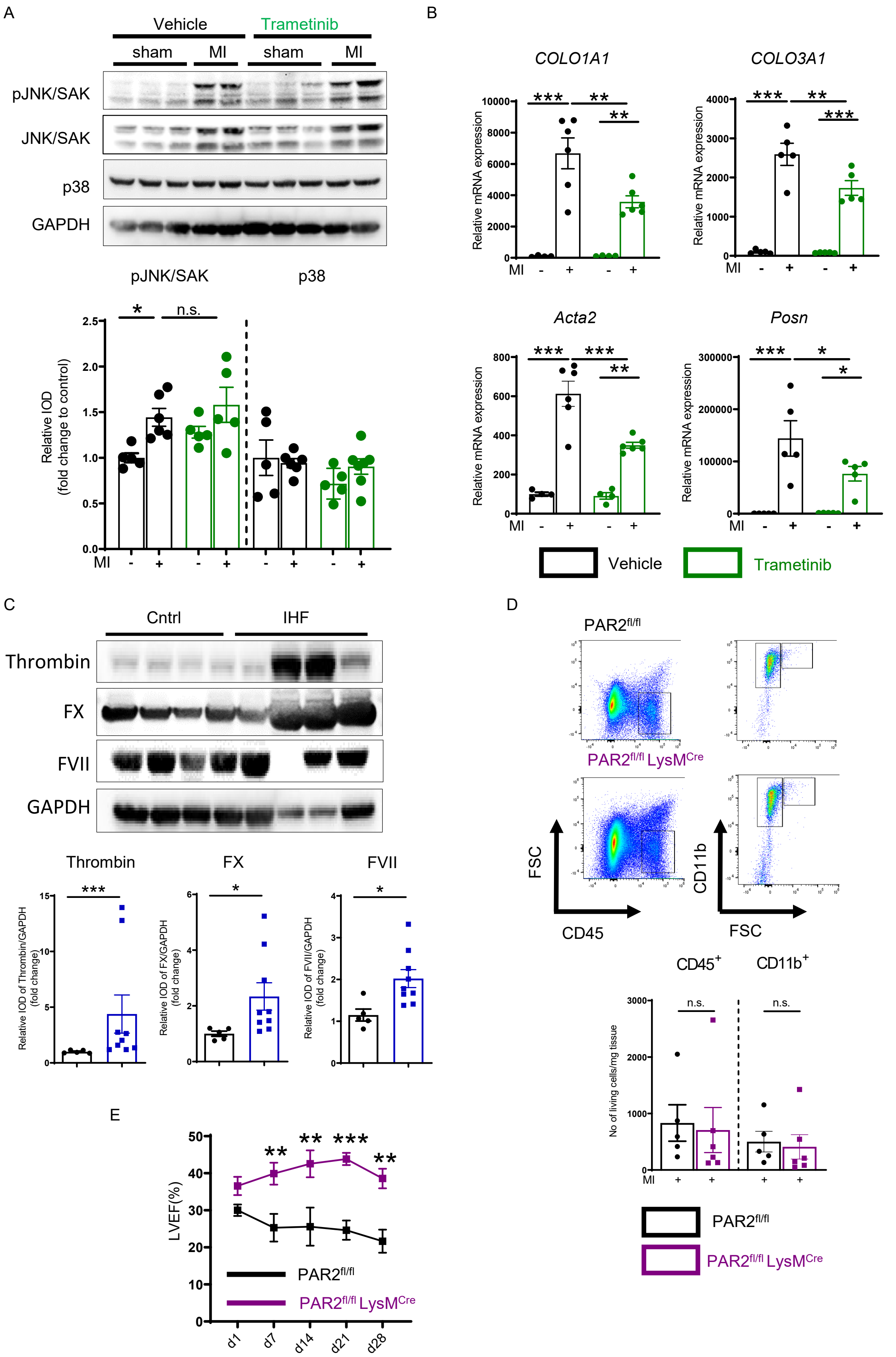
A
Vehicle
Trametinib
sham
MI
sham
MI
pJNK/SAK
JNK/SAK
p38
GAPDH
pJNK/SAK
p38
*
n.s.
Relative IOD (fold change to control)
MI
B
COLO1A1
COLO3A1
Relative mRNA expression
Acta2
Posn
Vehicle
Trametinib
C
Cntrl
IHF
Thrombin
FX
FVII
GAPDH
Relative IOD of Thrombin/GAPDH (fold change)
Relative IOD of FX/GAPDH (fold change)
Relative IOD of FVII/GAPDH (fold change)
D
PAR2fl/fl
PAR2fl/fl LysMCre
FSC
CD45
CD11b
FSC
CD45+
CD11b+
No of living cells/mg tissue
n.s.
PAR2fl/fl
PAR2fl/fl LysMCre
E
LVEF(%)
PAR2fl/fl
PAR2fl/fl LysMCre
d1
d7
d14
d21
d28

Supplementary Figure 12. A) Protein expression analysis of pJNK/SAK (normalized to total JNK/SAK) and p38 (normalized to GAPDH) in infarcted myocardium obtained from vehicle or trametinib treated mice; n = 4-6; one-way ANOVA, Sidak's multiple comparison test. **B)** Relative mRNA expression analysis of *COLOA1, COLO3A1, Acta2* and *Posn* from the infarcted myocardium obtained from vehicle or trametinib treated animals; n = 4-6; one-way ANOVA, Sidak's multiple comparison test. **C)** Western blot analysis for thrombin, FX, and FVII normalized to GAPDH and quantification of human left ventricular tissue obtained from n = 5 non-ischemic (NI) donor hearts and n = 9-10 IHF patients; Mann-Whitney test. **D)** Flow cytometry analysis of the infarcted myocardium obtained from $PAR2^{fl/fl}$ and $PAR2^{fl/fl}$ $LysM^{Cre}$ littermates at day 7. Representative dot plots and quantification of $CD45^{+}$ leukocytes and $CD45^{+}/CD90.2^{-}/NK1.1^{-}/CD11b^{+}$ myelomonocytic cells; n = 5; Mann-Whitney test. **E)** Echocardiographic studies of $PAR2^{fl/fl}$ and $PAR2^{fl/fl}$ littermates over 4 weeks for LVEF (%) in PLAX M-mode; n = 5-7; two-way ANOVA, Bonferroni's multiple comparisons test. Data are shown as mean ± SEM.

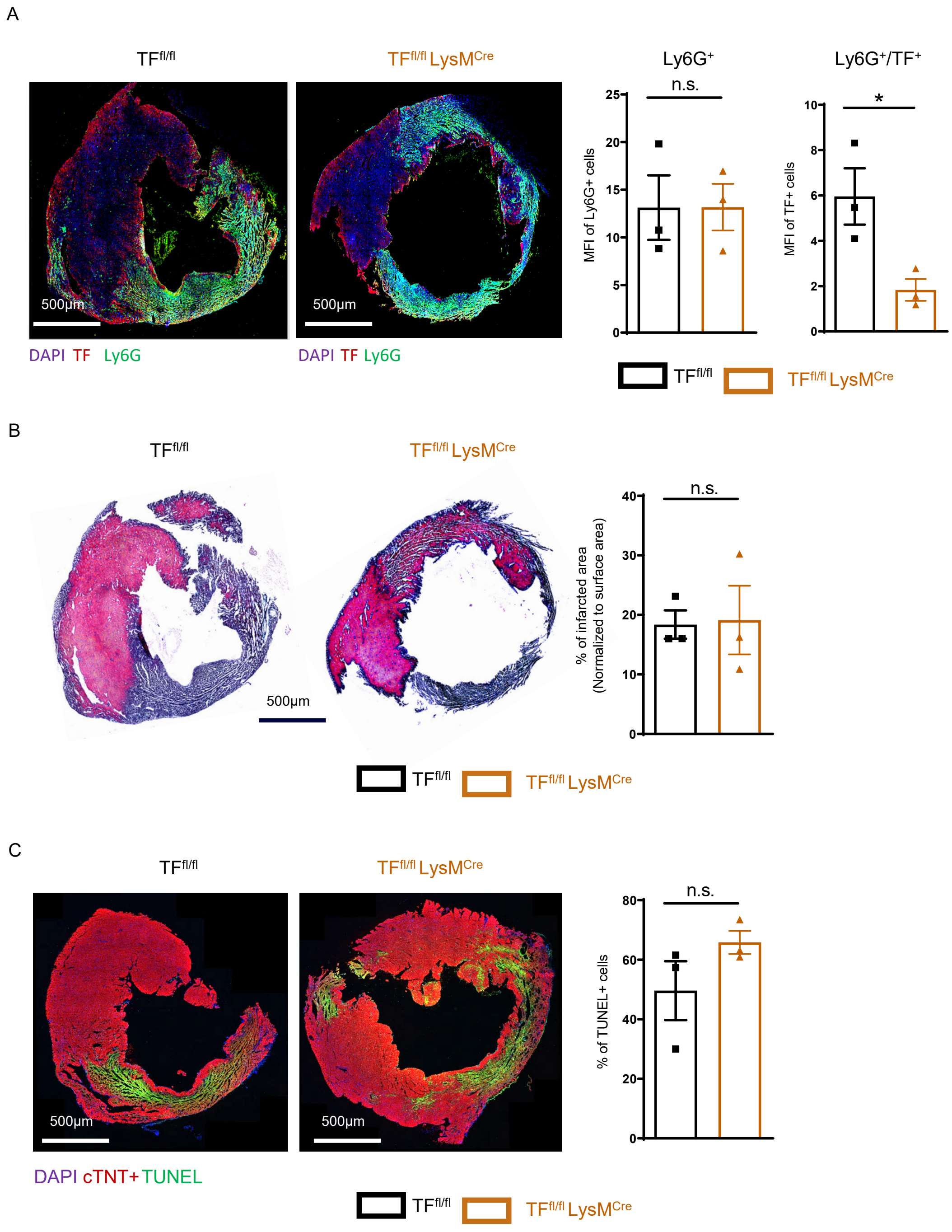

Supplementary Figure 13. A) Representative confocal images and quantification of Ly6G⁺/TF⁺ cells on myocardial cross-sections of $TF^{fl/fl}$ and $TF^{fl/fl}$ $LysM^{Cre}$ littermates at day 1; counterstained for Ly6G, TF and DAPI. **B)** Masson-Trichrome staining for infarct size assessment. Representative images and quantification. **C)** Representative confocal images and quantification of $cTNT^+$/$TUNEL^+$ cells in the infarcted myocardium; n = 3; Mann-Whitney test. Data are shown as mean ± SEM.

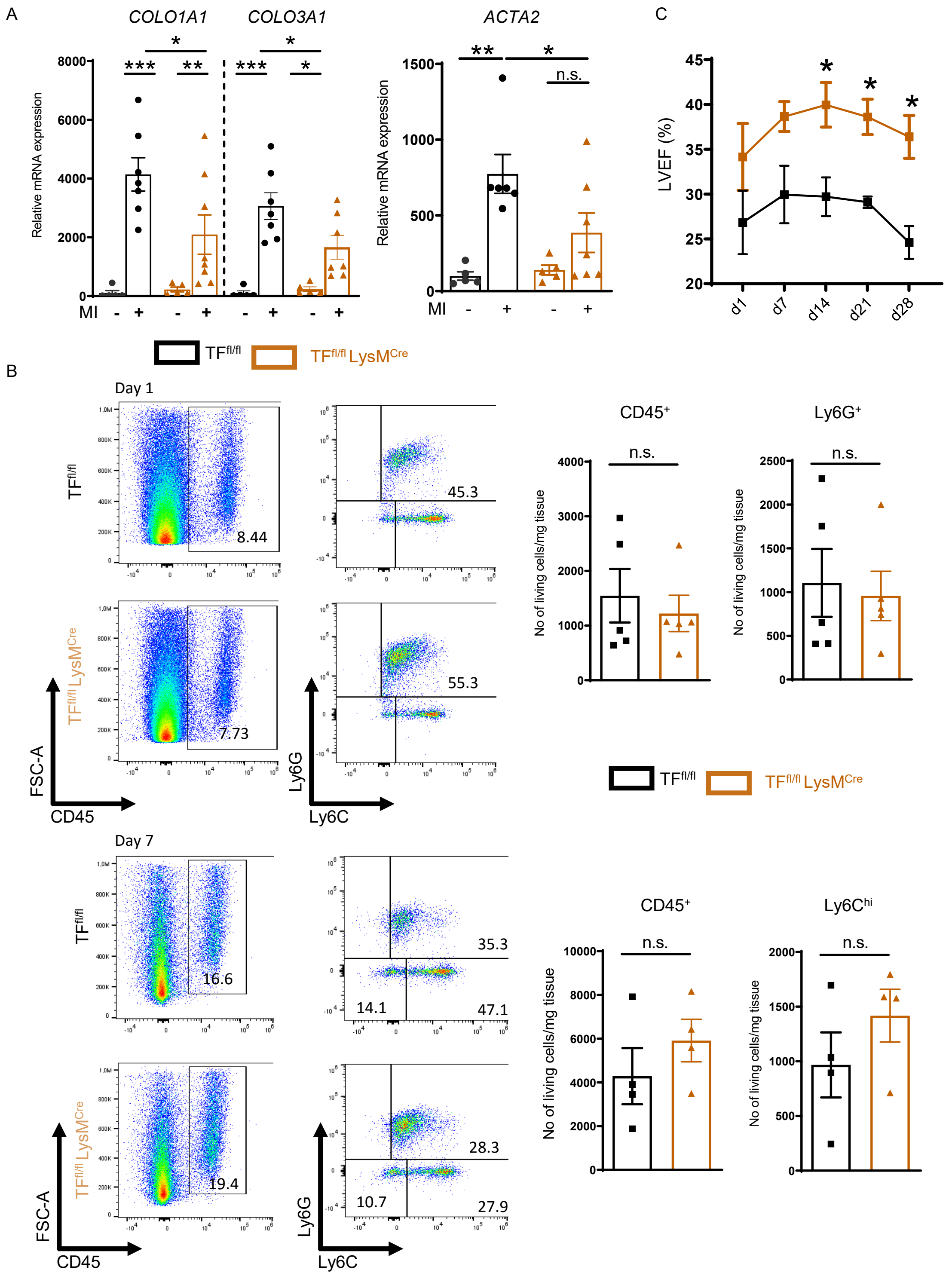
A
COLO1A1
COLO3A1
ACTA2
Relative mRNA expression
MI
C
LVEF (%)
d1
d7
d14
d21
d28
TFfl/fl
TFfl/fl LysMCre
B
Day 1
Day 7
FSC-A
CD45
Ly6G
Ly6C
CD45+
Ly6G+
Ly6Chi
n.s.
No of living cells/mg tissue

Supplementary Figure 14. A) Relative mRNA expression of *COLOA1, COLO3A1* and *Acta2* in the infarcted myocardium of $TF^{fl/fl}$ and $TF^{fl/fl}$ $LysM^{Cre}$ littermates; n = 5-7; one-way ANOVA, Sidaks multiple comparison test. **B)** Representative plots and quantification of $CD45^+$ and $CD45^+/CD90.2^-/NK1.1^-/CD11b^+/Ly6G^+$ neutrophils at day 1 and $CD45^+$ and $CD45^+/CD90.2^-/NK1.1^-/CD11b^+/Ly6G^+/Ly6C^{hi}$ monocytes at day 7; n = 4. Mann-Whitney test. Data are shown as mean ± SEM. **C)** Longitudinal echocardiographic studies of LVEF (%) in PLAX M-mode over 4 weeks; n = 7-8; two-way ANOVA, Bonferroni's multiple comparisons test**.** Data are shown as mean ± SEM.

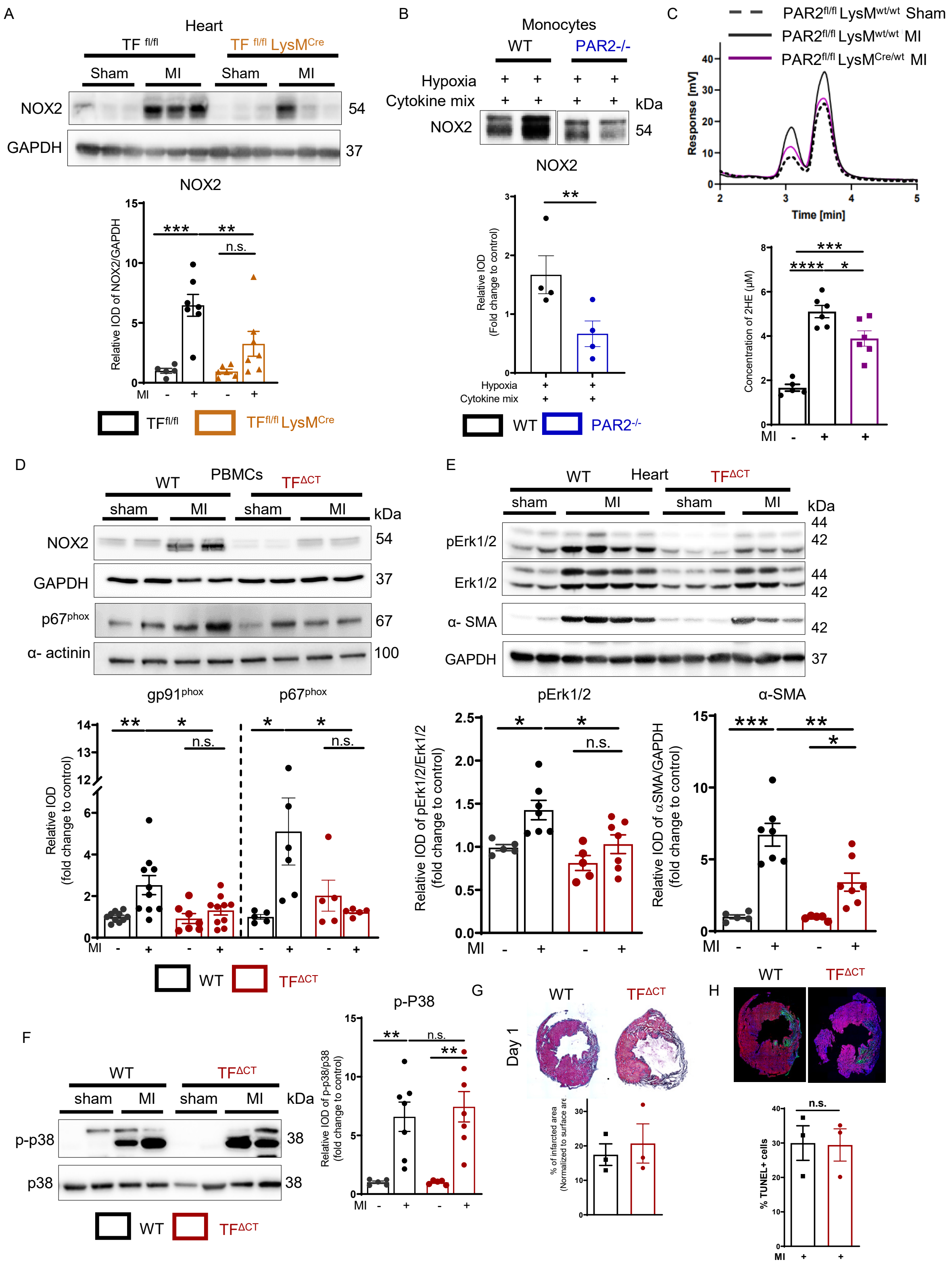
A
Heart
TF fl/fl
TF fl/fl LysMCre
Sham
MI
Sham
MI
NOX2
GAPDH
54
37
NOX2
Relative IOD of NOX2/GAPDH
***
**
n.s.
MI
TFfl/fl
TFfl/fl LysMCre
B
Monocytes
WT
PAR2-/-
Hypoxia
Cytokine mix
kDa
NOX2
54
NOX2
**
Relative IOD (Fold change to control)
Hypoxia
Cytokine mix
WT
PAR2-/-
C
PAR2fl/fl LysMwt/wt Sham
PAR2fl/fl LysMwt/wt MI
PAR2fl/fl LysMCre/wt MI
Response [mV]
Time [min]
***
****
*
Concentration of 2HE (µM)
MI
D
PBMCs
WT
TFΔCT
sham
MI
sham
MI
kDa
NOX2
GAPDH
p67phox
α- actinin
54
37
67
100
gp91phox
p67phox
Relative IOD (fold change to control)
**
*
n.s.
MI
WT
TFΔCT
E
Heart
WT
TFΔCT
sham
MI
sham
MI
kDa
pErk1/2
Erk1/2
α- SMA
GAPDH
44
42
37
pErk1/2
α-SMA
Relative IOD of pErk1/2/Erk1/2 (fold change to control)
Relative IOD of αSMA/GAPDH (fold change to control)
***
**
*
n.s.
MI
F
WT
TFΔCT
sham
MI
sham
MI
kDa
p-p38
p38
38
p-P38
Relative IOD of p-p38/p38 (fold change to control)
**
n.s.
MI
WT
TFΔCT
G
WT
TFΔCT
Day 1
% of infarcted area (Normalized to surface area)
H
WT
TFΔCT
% TUNEL+ cells
n.s.
MI

Supplementary Figure 15. A) Protein expression analysis of NOX2 (normalized to GAPDH) in the infarcted myocardium of $TF^{fl/fl}$ and $TF^{fl/fl}$ $LysM^{Cre}$ littermates. Loading control is identical to Fig. 7B; one-way ANOVA; Sidaks multiple comparison test; and **B)** of monocytes isolated from $PAR2^{-/-}$ animals (loading control from Figure 4B). Ordinary one-way ANOVA, Sidak's multiple comparison test; n = 5 samples per group (2-3 mice were pooled for each sample). **C)** Assessment of superoxide formation in infarcted myocardium of $PAR2^{fl/fl}$ and $PAR2^{fl/fl}$ $LysM^{Cre}$ at day 7 by DHE-HPLC. Representative chromatogram of 2-HE, the oxidation product of DHE, and quantification normalized to total protein counts; one-way ANOVA, Sidak,s multiple comparison test. **D)** Western blot analysis of $gp91^{phox}$ and $p67^{phox}$ (normalized to α-actinin) expressed in PBMCs of the experimental animals. Representative blots and quantification of biological replicates; one-way ANOVA and Sidak's multiple comparison test. **E)** Western blot analysis of pERK1/2 (normalized to total ERK1/2), α-SMA (normalized to GAPDH) in the infarcted myocardium obtained from WT or $TF^{\Delta CT}$ mice 7 days after MI**.** Representative blots and quantification of biological replicates; one-way ANOVA, Sidak's multiple comparison test. **F)** Protein expression analysis of pP38 normalized to total P38 in the infarcted myocardium obtained from WT or $TF^{\Delta CT}$ mice 7 days after MI; one-way ANOVA, Sidak's multiple comparison test. Masson-Trichrome staining for infarct size assessment **G)** and representative confocal images and quantification of $cTNT^{+}$/$TUNEL^{+}$ cells **H)** of WT and $TF^{\Delta CT}$ mice 1 day after permanent LAD ligation. Data are shown as mean ± SEM.

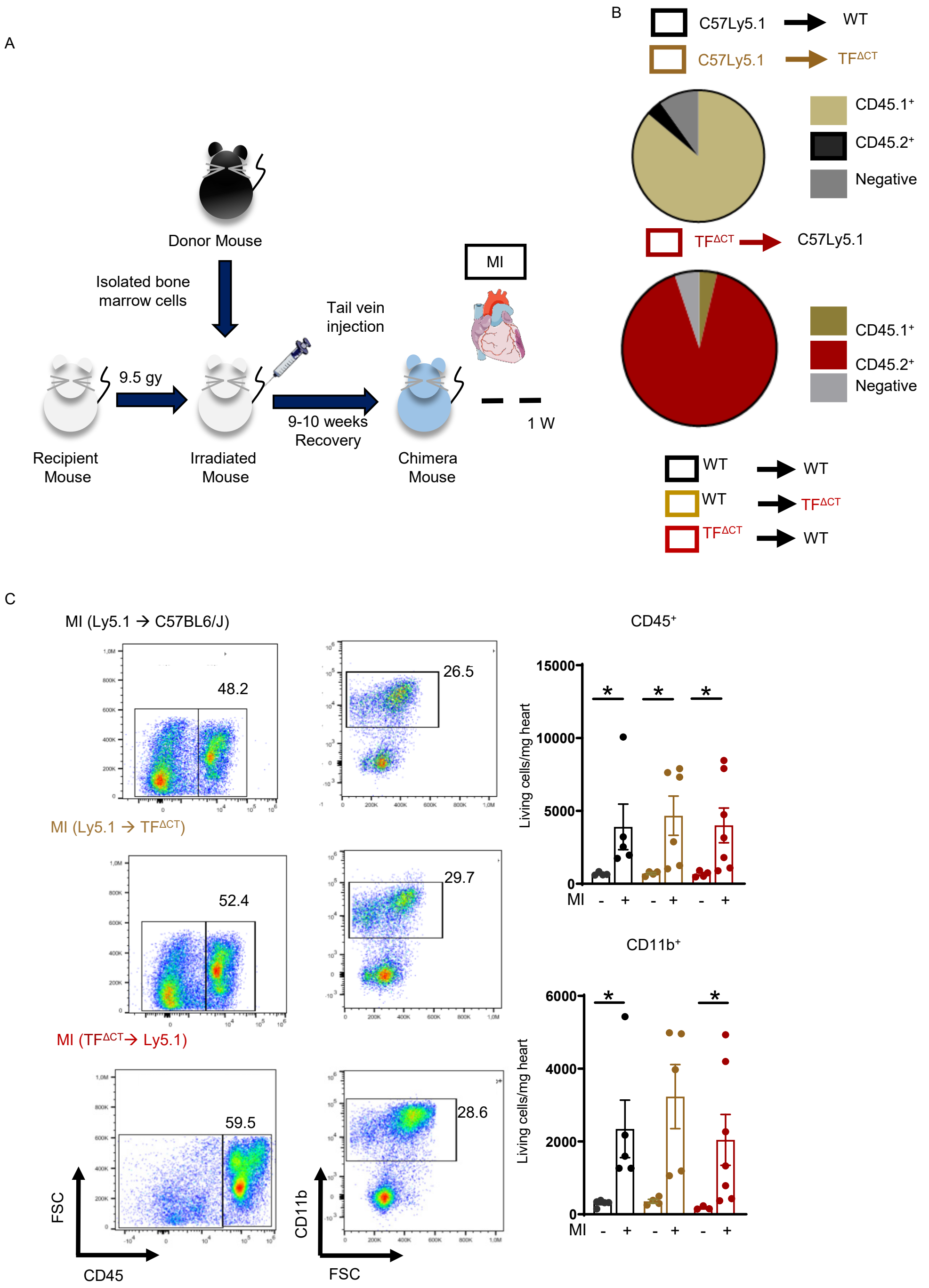

Supplementary Figure 16. A) Scheme of the BM transplantation experiment. **B)** Representative pie chart showing the percentage of peripheral blood CD45.1^{+} and CD45.2^{+} cells to quantify donor chimerism. **C)** Flow cytometry analysis of the infarcted myocardium obtained from BM transplanted mice. Representative dot plots and quantification of CD45^{+} leukocytes and CD45^{+}/CD90.2^{-}/NK1.1^{-}/CD11b^{+} myelomonocytic cells; n= 4-5; Kruskal-Wallis test and Dunns-multiple comparison test. Data are shown as mean ± SEM.

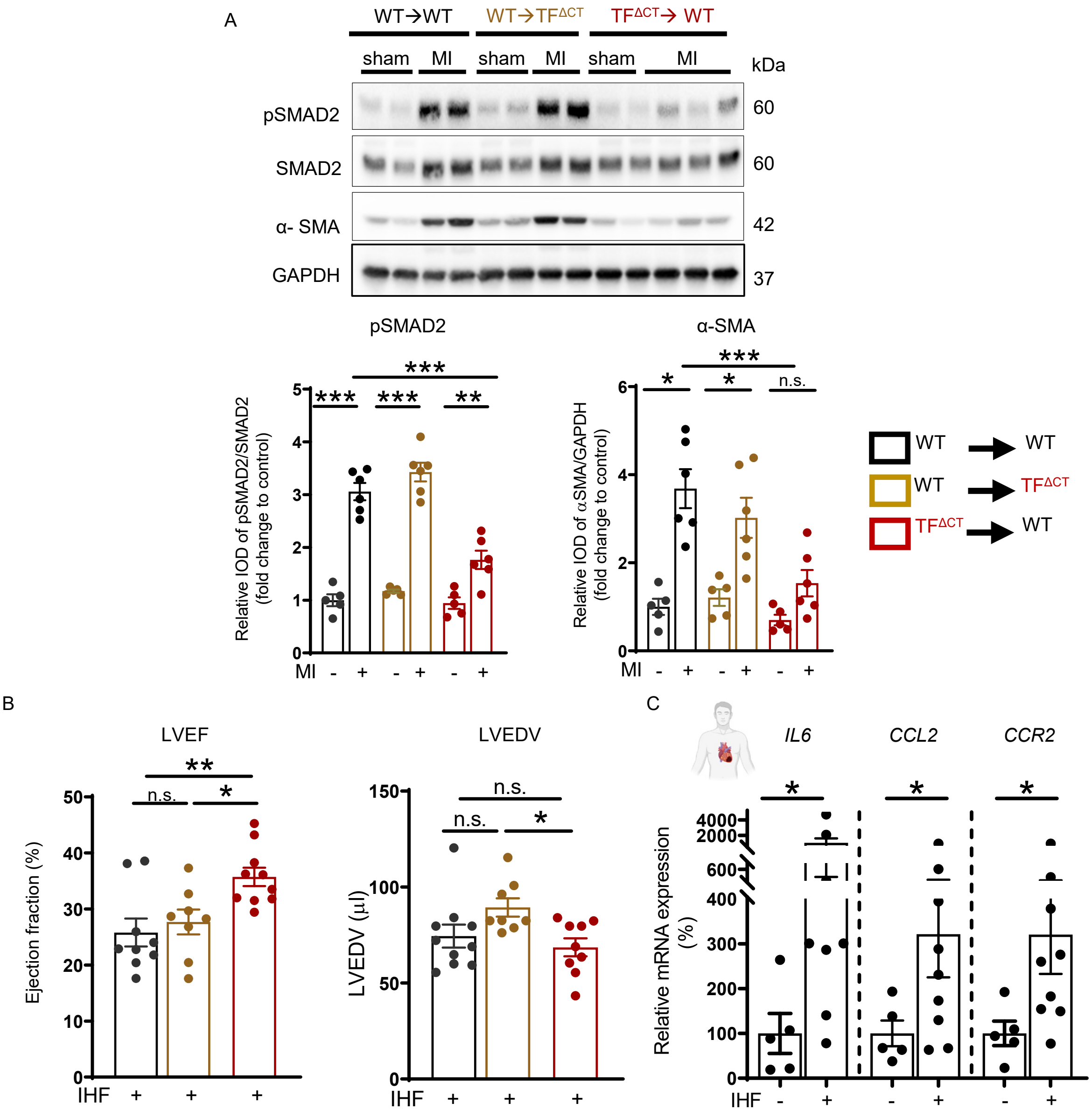

Supplementary Figure 17. A) Western blot analysis of pSMAD2 (normalized to SMAD2) and α-SMA (normalized to GAPDH) in infarcted myocardium obtained from BM transplanted mice at day 7 post MI. Representative blots and quantification of biological replicates; one-way ANOVA, Sidak's multiple comparison test. **B)** LVEF (%) and LVEDV (µl) in PLAX M-mode 6 weeks after permanent LAD ligation; one-way ANOVA, Sidak's multiple comparison test. **C)** mRNA expression analysis of *IL6*, *CCL2* and *CCR2* in human left ventricular tissue obtained from the n = 5 non-infarct donor (NI) and n = 9 patient (IHF) hearts; Mann-Whitney test.

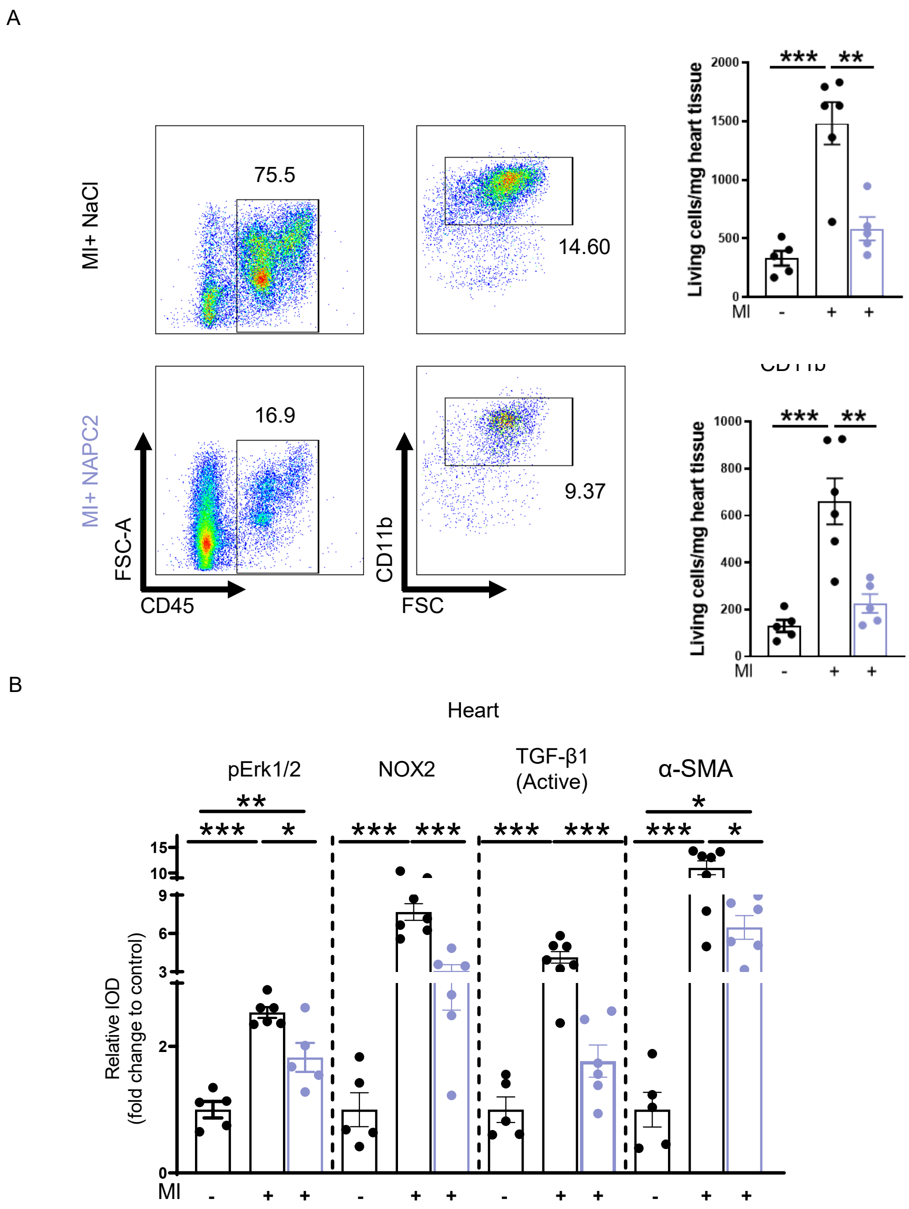

Supplementary Figure 18. A) Flow cytometric analysis of infarcted myocardium obtained from NAPc2 treated animals (1 mg/kg/d) normalized to heart weight. Representative dot plots and quantification of $CD45^+$ and $CD45^+/CD90.2^-/NK1.1^-/CD11b^+$ leukocytes; one-way ANOVA, Sidaks multiple comparison test. **B)** Quantification of pERK1/2 (normalized to total ERK1/2), NOX2, TGF-β1 and α-SMA (normalized to GAPDH) of representative Western Blots shown in Figure 8E.

Supplementary Tables:

Supplementary table 1: Raw data of the proteomics data set. See linked Excel file uploaded as separated supplementary table.

Supplementary table 2: Raw data of the phospho-proteomics data set. See linked Excel file uploaded as separated supplementary table.

Supplementary table 3: Individual proteomic and phosphor proteomic intensities of complement and coagulation hits with exact adjusted P value. See linked Excel file uploaded as separated supplementary table.

#	Diagnosis	OP	sex	Age	EF (%)[a]	cTNI (µg/l)[b]
IHF 1	IHF	LVAD	m	63	18	n.a.
IHF 2	IHF	LVAD	f	49	10	148
IHF 3	IHF	TAH	m	73	<10	330
IHF 4	IHF	TAH	m	40	49	>50
IHF 5	IHF	TAH	m	45	15	162
IHF 6	IHF	TAH	m	58	20	179
IHF 7	IHF	TAH	m	48	20	138
IHF 8	IHF	TAH	m	45	10	48.9
IHF 9	IHF	TAH	m	47	15	127
IHF 10	IHF	TAH	m	63	15	160
Donor 1	NI	EXPL	f	40	-	-
Donor 2	NI	EXPL	m	61	-	-
Donor 3	NI	EXPL	f	64	-	-
Donor 4	NI	EXPL	m	44	-	-
Donor 5	NI	EXPL	f	54	-	-

Supplementary Table 4. Patient Characteristics of patients with severe ischemic heart failure compared to age-matched donors (mean age ± SEM: 52.0 ± 3.5 vs. 52.6 ± 4.6 years, $p = 0.95$). Human heart samples were obtained during total artificial heart implantation (TAH) and left ventricular assistant device implantation (LVAD) from patients with ischemic heart failure (IHF), or from donor hearts (D) declined for transplantation (explanted non-ischemic hearts (EXPL). Samples from patients #3 to #7 as well as #11 to #15 were randomly assigned to the (phospho)proteomic study. m, male; f, female; EF, ejection fraction; cTNI, cardiac troponin I; n.a., not available. [a, b] Donor hearts were only accepted when EF was > 55% and troponin levels were negative. A stratum of the IHF samples was randomly selected, labeled samples # IHF 1-5, and compared to samples # D 1-5 to perform the (phospho)proteomics analyses in quadruplictes, as depicted in Figure 1.

	**CAD †**	**Subacute MI ††**	
n	6	6	n.s.
age (years)	78 ± 2	70 ± 6	n.s.
Male, n (%)	6 (100%)	5 (84%)	n.s.
History of smoking, n (%)	4 (66%)	1 (16%)	p = 0.0357
BMI (Kg/m^2)	28.6 ± 1.1	26.6 ± 2.52	n.s.
Heart Rate, bpm	67.2 ± 5.1	79.8 ± 7.6	n.s.
Alcohol Consumption, n (%)	0%	0%	n.s.
History of Diabetes mellitus, n (%)	1 (16%)	0 (0%)	n.s.
History of Peripheral Artery Disease, n (%)	0 (0%)	0 (0%)	n.s.
Ejection Fraction (EF%)	47 ± 8.4	44 ± 11.4	n.s.
WBC (cells/µl)	9866 ± 985	8033 ± 844	n.s.
Neutrophils (cells/µl)	6983 ± 980	6150 ± 950	n.s.
Platelets (cells/µl)	230500 ± 16059	252333 ± 36010	n.s.
Cardiac Troponin I Levels (pg/ml)	34.35 ± 7.85	49034 ± 21602	p = 0.0357
CRP (mg/l)	4.76 ± 1.50	81.2 ± 25.7	p = 0.0114
Creatinine Kinase (U/l)	129.2 ± 33.45	1285 ± 557.04	p = 0.0442
Serum Creatinine (mg/dl)	1.18 ± 0.19	1.43 ± 0.23	n.s.
**Medication n (%)**			

Aspirin	6 (100%)	6 (100%)	n.s.
ACE inhibitors	2 (32%)	4 (66%)	n.s.
AT 1 receptor blockers	1 (16%)	2 (32%)	n.s.
β-blockers	6 (100%)	4 (66%)	n.s.
Statins	5 (84%)	5 (84%)	n.s.
P2Y12 inhibitors	6 (100%)	6(100%)	n.s.
Anti-Coagulation Treatment (Heparin, Vitamin-K agonists)	3 (50%)	4 (66%)	n.s.
GPIIb/IIIa inhibitors	4 (67%)	2 (33%)	n.s.
Major Adverse Cardiovascular Events	0 (0%)	0 (0%)	n.s.

Supplementary Table 5. Patient characteristics selected from the MICAT registry. We included patients that were subjected to percutaneous coronary intervention with either stable coronary artery disease (CAD) or patients with subacute MI. All participants had been enrolled in the MICAT registry (Mainz Intracoronary database, ClinicalTrials.gov Identifier: NCT02180178). †, 100% had 3-vessel CAD; ††, 50% had 3-vessel-CAD, 50% had 2-vessel-CAD, 0% had 1-vessel CAD, n.s. Chi-square test. BMI, body mass index; WBC, white blood cell count; CRP, C-reactive protein; n.s., not significant.
